# Implementation and Calibration of a Deep Neural Network to Predict Parameters of Left Ventricular Systolic Function Based on Pulmonary and Systemic Arterial Pressure Signals

**DOI:** 10.3389/fphys.2020.01086

**Published:** 2020-09-11

**Authors:** Jean Bonnemain, Luca Pegolotti, Lucas Liaudet, Simone Deparis

**Affiliations:** ^1^Adult Intensive Care and Burn Unit, University Hospital and University of Lausanne, Lausanne, Switzerland; ^2^SCI-SB-SD, School of Basic Sciences, Ecole Polytechnique Fédérale de Lausanne, Institute of Mathematics, Lausanne, Switzerland

**Keywords:** heart failure, hemodynamics, deep neural network, cardiovascular modeling, blood flow model, machine learning

## Abstract

The evaluation of cardiac contractility by the assessment of the ventricular systolic elastance function is clinically challenging and cannot be easily obtained at the bedside. In this work, we present a framework characterizing left ventricular systolic function from clinically readily available data, including systemic and pulmonary arterial pressure signals. We implemented and calibrated a deep neural network (DNN) consisting of a multi-layer perceptron with 4 fully connected hidden layers and with 16 neurons per layer, which was trained with data obtained from a lumped model of the cardiovascular system modeling different levels of cardiac function. The lumped model included a function of circulatory autoregulation from carotid baroreceptors in pulsatile conditions. Inputs for the DNN were systemic and pulmonary arterial pressure curves. Outputs from the DNN were parameters of the lumped model characterizing left ventricular systolic function, especially end-systolic elastance. The DNN adequately performed and accurately recovered the relevant hemodynamic parameters with a mean relative error of less than 2%. Therefore, our framework can easily provide complex physiological parameters of cardiac contractility, which could lead to the development of invaluable tools for the clinical evaluation of patients with severe cardiac dysfunction.

## 1. Introduction

Heart failure corresponds to a clinical syndrome with a wide spectrum of symptoms, ranging from dyspnea and exercise intolerance to cardiogenic shock. It is caused by structural or functional cardiac abnormalities that result in low cardiac output, i.e., the inability of the heart to provide sufficient blood flow to satisfy the metabolic needs of the organism (Metra and Teerlink, 2017). It affects approximately 2% of the population, with a lifetime risk of developing heart failure of 20%, and a 5-years mortality of about 50% (Yancy et al., 2013). In the intensive care unit (ICU), cardiogenic shock represents 6% of admission, with an in-ICU mortality as high as 50% (Puymirat et al., 2017).

Evaluation of cardiac function is of crucial importance since it directly impacts treatment and therefore prognosis. In particular, the early and repeated evaluation of the left ventricular function is a key factor to guide therapies. This is particularly relevant in the ICU setting, where hemodynamic changes in unstable patients can occur rapidly (minutes). The aim of hemodynamic monitoring is therefore to accurately and rapidly identify changes in order to adapt treatment.

Hemodynamic assessment of cardiogenic shock is nowadays multimodal and includes clinical evaluation and paraclinical examination. Echocardiography for this situation is currently the cornerstone tool since it provides a complete evaluation of cardiac function and structure and can also predict response to treatment, in particular fluid-responsiveness (Vieillard-Baron et al., 2019). However, this examination is time- and resources-consuming (although new sensor technology may reduce the costs of these devices), hence evaluation based only on echocardiography may result in delays in management. Moreover, it cannot be used in a continuous manner. Other methods, either invasive or non-invasive, allow to indirectly assess cardiac output and volumes (Alhashemi et al., 2011; Monnet and Teboul, 2017; Nguyen and Squara, 2017).

Although these tools provide accurate estimates of ventricular function, evaluation of cardiac mechanics is fully characterized using the measure of pressure and volume of the ventricle during the entire cardiac cycle. From these values one can establish the ventricular pressure-volume diagram and determine the end-systolic and end-diastolic pressure-volume relationships. The former permit the determination of ventricular end-systolic elastance (Ees), a load-independent measurement of ventricular contractility (Burkhoff et al., 2005; Naeije and Manes, 2014). Nowadays these measurements require invasive techniques only available in the catheterization laboratory (Bonnet et al., 2017). Non-invasive methods have been developed, including magnetic resonance imaging (Bastos et al., 2019), or real-time three dimensional echocardiography (Seemann et al., 2019), but these cannot be easily implemented in the daily clinical practice.

To circumvent the drawbacks of these different methods, we aimed at developing a framework to characterize parameters of ventricular systolic function from easily accessible clinical data, namely systemic and pulmonary arterial pressures. The gold standard for such measurements is the use of invasively inserted intravascular catheters in the radial or femoral artery (systemic arterial pressure), or the pulmonary artery (pulmonary artery pressure). To obtain accurate pressure values, the measurements must be done at end-expiration and in the supine position, with standard zero reference at the level of the right atrium. Some variability of intravascular pressure measurement may still occur as a consequence of over- or underdamping of the signal (Romagnoli et al., 2014). We implemented and calibrated a deep neural network (DNN)—trained with data obtained from a lumped model of the cardiovascular system (Shi et al., 2011), as developed by Ursino (1998)—to model different levels of severity of left ventricular systolic dysfunction. This DNN takes, as input, systemic and pulmonary arterial pressure signals and returns, as output, the parameters of the lumped model that characterize left ventricular function, in particular Ees. The inputs are relevant measurable clinical values, whereas the predicted outputs—as mentioned above—cannot be easily evaluated in clinical practice; these are in turn provided to the 0D model in order to recover all other clinical values, especially the ventricular pressure-volume curves.

The role of the proposed DNN is to solve an inverse problem which maps some of the outputs of the 0D model (i.e., systemic and pulmonary arterial pressures) to its underlying parameters. Of note, previous investigators, (Pennati et al., 1997; Pant et al., 2016; Schiavazzi et al., 2017) have proposed alternate methods for parameter identification of lumped parameter models in other physiological conditions, such as single-ventricle physiology with valve dysfunction (Pant et al., 2016). In this paper, we present a novel deep-learning-based approach to solve this issue.

## 2. Materials and Methods

### 2.1. Global Framework

**Figure 1:**
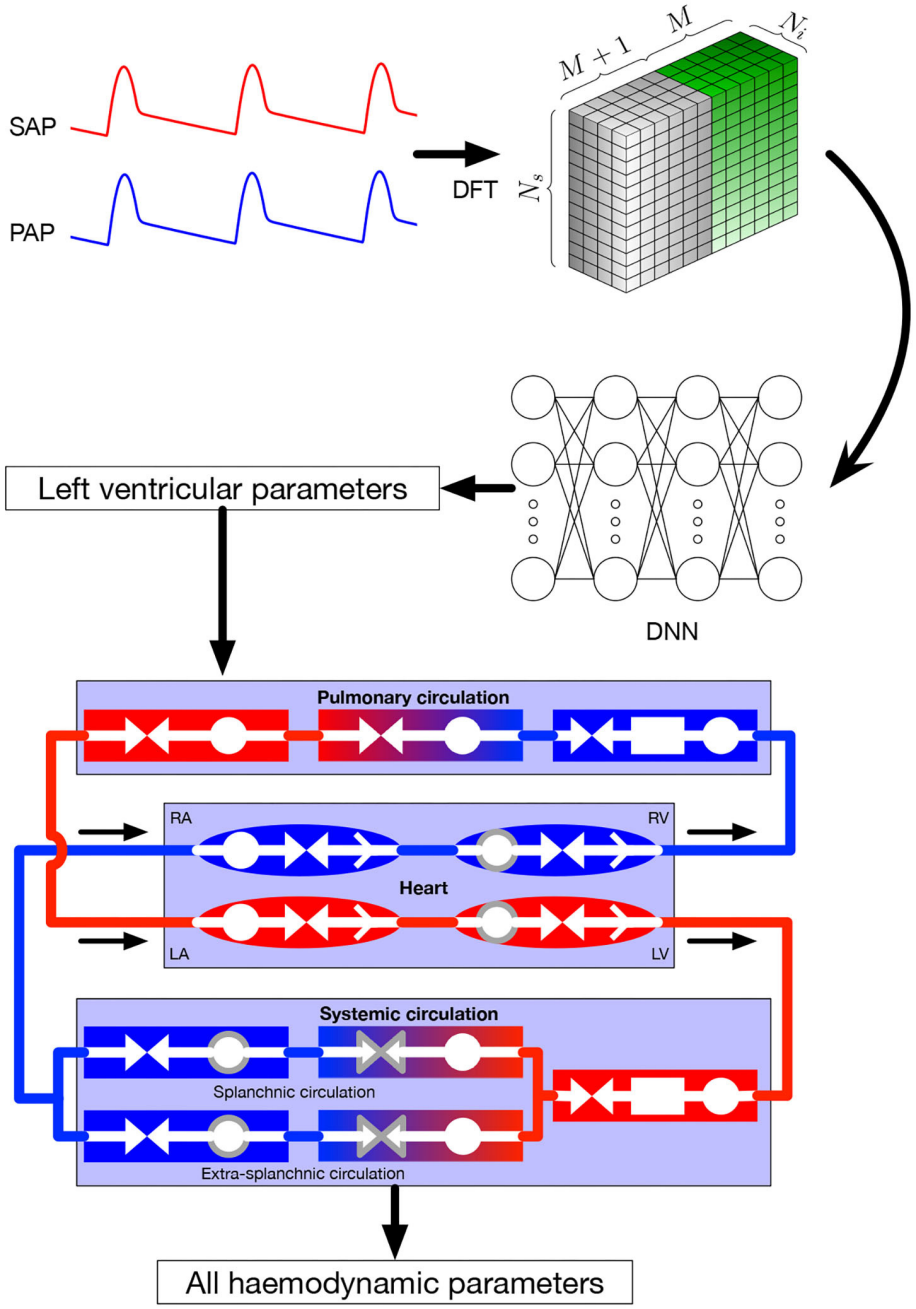
Global framework. Input parameters, systemic and pulmonary arterial curves are represented in the frequency domain and then given to the DNN, that itself returns the left ventricular parameters of the 0D model. These parameters are given to the 0D model, allowing to recover numerous hemodynamic values. Symbols in the 0D model are: circles: compliance; facing triangles (double arrows): resistance; rectangles: inertance; single white arrows in heart chambers: cardiac valves; gray contour: elements affected by autoregulation; red elements: oxygenated blood, blue elements: deoxygenated blood. SAP, systemic arterial pressure; PAP, pulmonary arterial pressure; DFT, discrete Fourier transform, providing the coefficients *a*_*k*_ and *b*_*k*_; DNN, deep neural network; RA, right atrium; RV, right ventricle; LA, left atrium; LV, left ventricle.

In this section, we focus on the online pipeline of our method (i.e., the prediction procedure) which is depicted in Figure 1, whereas the training process is described in section 2.3 for general DNNs and section 2.4 for our specific application. Input parameters were systemic and pulmonary arterial pressure curves. Theses curves are represented in the frequency domain by their Fourier coefficients. As explained in more detail in section 2.4, the Fourier coefficients were subsequently arranged in a three-dimensional tensor which served as input for the DNN. The output of the DNN were the four parameters characterizing left ventricular function in the 0D model, namely *E*_max,lv_, *E*_max_lv,0__, *G*_*E*_max,lv__, and *k*_E,lv_ (see section 2.2 for more details). In the last step, the predicted parameters were provided to the 0D model to simulate and recover all the other values.

### 2.2. Lumped Parameters Model of the Cardiovascular System

The lumped model of the cardiovascular system has been extensively described in the original paper by Ursino (1998). The model provides a mathematical description of the entire cardiovascular system with time-varying elastance of the left and right ventricles. It also includes the afferent carotid baroreceptor pathway, the sympathetic and vagal efferent activities, the splanchnic and extrasplanchnic systemic circulation, and the pulmonary circulation. A representation of the model is presented at the bottom of Figure 1. For each of the vascular *i*-compartment, mass and momentum conservation are given by, respectively:

(1) $$\begin{array}{r} Q_{\text{in}}\left(t\right)-Q_{\text{out}}\left(t\right)=\frac{\text{d}V_{\text{i}}\left(t\right)}{\text{d}t}, \end{array}$$

and

(2) $$\begin{array}{r} P_{\text{in}}\left(t\right)-P_{\text{out}}\left(t\right)=R_{\text{i}}Q_{\text{out}}\left(t\right)+L_{\text{i}}\frac{\text{d}Q_{\text{out}}\left(t\right)}{\text{d}t}, \end{array}$$

where *V*_i_ is the volume of the *i*-compartment, *Q*_in_ and *Q*_out_ the inflow and outflow rate, *P*_in_ and *P*_out_ the inlet and outlet pressure, *R*_i_ the resistance, and *L*_i_ the inertance. The pressure and flow relationship reads

(3) $$\begin{array}{r} \frac{\text{d}P_{\text{in}}\left(t\right)}{\text{d}t}=\frac{1}{C_{\text{i}}}\frac{\text{d}V_{\text{i}}\left(t\right)}{\text{d}t}. \end{array}$$

Inertance is taken into account for arterial compartments where acceleration of blood is significant, while neglected for the other ones. Valve opening is determined by its pressure gradient across the valve, i.e., it opens when *P*_in_ > *P*_out_. Left and right ventricle contractility is modeled as a time-varying elastance. Efferent pathways act on several parameters, namely heart period, left and right ventricle contractility, unstressed volumes (defined as the volume when pressure is equal to zero), and both splanchnic and extrasplanchnic peripheral artery resistance. Parameters affected by autoregulation are surrounded by a gray line in Figure 1.

In a previous work (Bonnemain et al., 2013), we modified the model to account for left ventricular systolic failure at different levels of severity and validated it with clinical data. To reproduce various degrees of left ventricular systolic failure, we varied the following parameters:

- *E*_max,lv_ [mmHg/ml], reference value of the ventricle elastance,
- *E*_max_lv,0__ [mmHg/ml], reference value of the ventricle elastance in absence of autoregulation,
- *G*_*E*_max,lv__ [mmHg/ml/(spikes/s)], maximum baroreceptor gain,
- *k*_E,lv_ [1/ml], steepness of the pressure-volume curve.

**Figure 2:**
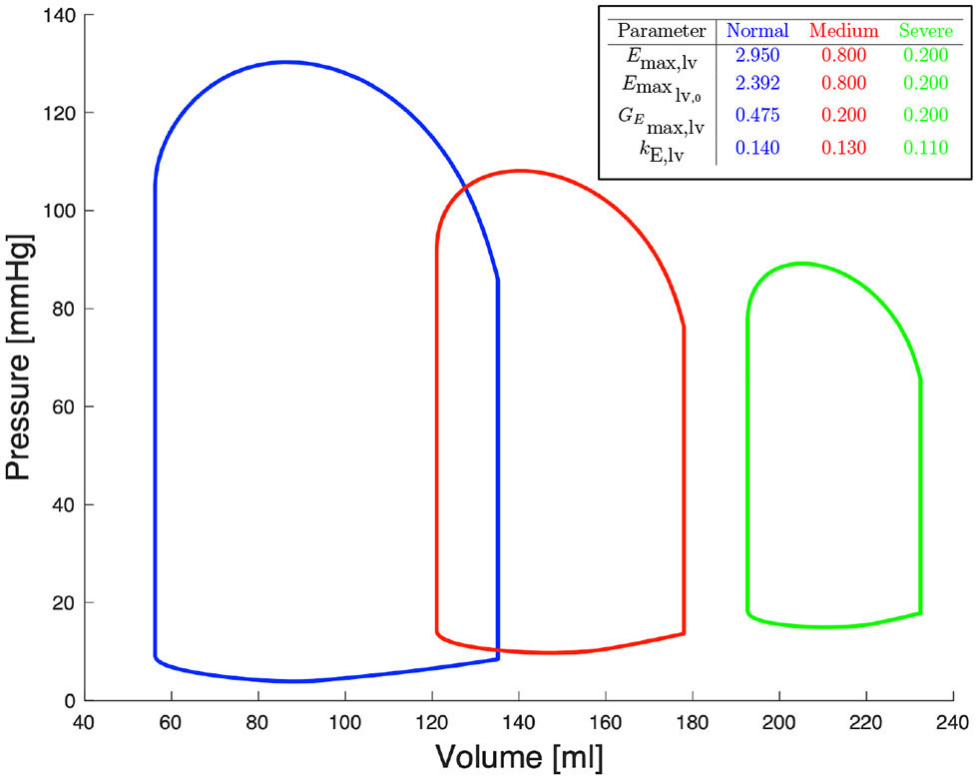
Pressure-volume diagrams for different degrees of systolic heart failure and their corresponding parameters in the 0D model.

Figure 2 shows left ventricular pressure-volume loops at different degrees of severity of heart failure. All other haemodynamic values are presented in Bonnemain et al. (2013). For each compartment, pressure, volume and flow can be extracted; it is therefore straight-forward to retrieve the ventricular pressure-volume curves.

### 2.3. Deep Neural Network

The term *machine learning* is typically used to indicate a family of algorithms and methods which, broadly speaking, are capable of identifying structures and patterns in complex and (usually) high-dimensional information. Due to the abundance of data obtained during the last decade and the ever increasing computational power of processors, graphics processing units (GPUs) and supercomputers, these methods have gained particular attention in all the fields of medicine (Komorowski et al., 2018; McKinney et al., 2020).

DNNs are a class of data-hungry machine learning algorithms notably suited for classification and regression tasks. They are used to approximate unknown mappings **f**(·) of the form **y** = **f**(**x**; ω), where **x** and **y** are input and output of the model, respectively, and ω indicates a set of parameters of the network. The function **f**(·), which, in reality, can be as general as possible, is represented in DNNs as a composition of easily computable parameterized functions **f**_1_, **f**_2_, …, **f**_*l*_ (where *l* is the number of layers of the network), i.e., **f** = **f**_*l*_ ◦ … ◦ **f**_2_ ◦ **f**_1_. In this work, we focused on the most basic type of DNNs, i.e., multi-layer perceptrons, which, despite their simple structure, have been mathematically proven to satisfy desirable approximation properties. In particular, multi-layer perceptrons with one hidden layer or more are universal approximators, see e.g., (Cybenko, 1988; Mhaskar, 1993). In contrast to more advanced architectures (e.g., convolutional neural networks), multi-layer perceptrons are solely based on a sequence of fully-connected layers of the form

(4) $$\begin{array}{r} {\text{y}}_{i}={\text{f}}_{i}\left({\text{x}}_{i};\omega_{i}=\left\{W_{i},{\text{b}}_{i}\right\}\right)=\sigma_{i}\left(W_{i}{\text{x}}_{i}+{\text{b}}_{i}\right), \end{array}$$

where the subscript *i* refers to the *i*th layer of the network, **x**_*i*_ = **y**_*i*−1_ is the corresponding input, *W*_*i*_ and **b**_*i*_ are a weight matrix and a bias vector, and σ_*i*_ is a non-linear activation function. Among the most common activation functions are e.g., the rectifier linear unit ReLU(*x*) = max(0, *x*), sigmoid(*x*) = 1/(1 + exp(−*x*)), and tanh(*x*). In this paper, we consider the ReLU and sigmoid activation functions for the hidden (i.e., **f**_1_, …, **f**_*l*−1_) and output (i.e., **f**_*l*_) layers, respectively. The weight matrix *W*_*i*_ and the bias vector **b**_*i*_ represent the parameters ω_*i*_ of the *i*th layer. In order to find an appropriate numerical value of the parameters ω_*i*_, for each *i* = 1, 2, …, *l*, the DNN has to be trained on a set of input-output pairs $Q=\left\{\left\{{\text{x}}^{1},{\text{y}}^{1}\right\},\left\{{\text{x}}^{2},{\text{y}}^{2}\right\},\ldots ,\left\{{\text{x}}^{n_{t}},{\text{y}}^{n_{t}}\right\}\right\}$ which constitute the so called training dataset. The ultimate goal of the training process is to find ω = {ω_1_, ω_2_, …, ω_*l*_} such that the DNN **f**(·, ω) performs a “good” fitting on *Q*, i.e., **f**(**x**^*k*^, ω) ≈ **y**^*k*^ for every *k* = 1, 2, …, *n*_*t*_. This is typically done by introducing a loss function acting on the training dataset $L_{\omega }\left(Q\right)$ and by minimizing it by means of optimization algorithms (usually, gradient or stochastic gradient descent). For a complete review of DNNs and their optimization, we refer the reader to Goodfellow et al. (2016).

### 2.4. The Offline Phase: Data Generation and Training of the Deep Neural Network

In the data generation phase, the 0D model described in section 2.2 is solved *N*_s_ times by randomly sampling *E*_max,lv_, *E*_max_lv,0__, *G*_*E*_max,lv__, and *k*_E,lv_. Specifically, we determined for each of these parameters a range of physiological or pathological (severe heart failure) values; for details about these ranges see (Bonnemain et al., 2013). Upper and lower bounds are reported in Figure 2. Each training sample was generated from the 0D model, by assigning a random individual value (drawn from a uniform distribution defined on the corresponding admissible range) for each of the four parameters of interest, and by keeping constant all the other parameters of the 0D model. Other parameters include values of resistance, compliance, unstressed volume, inertance, as well as values describing heart function, and values related to autoregulation, as presented in detail by Ursino (1998). This approach allows generating a dataset which includes a rich representation of all possible pathological conditions leading to left heart failure. Regarding sampling strategies, owing to the negligible computational cost required the generate each random sample, we did not consider alternative sampling strategies (e.g., Latin Hypercube or orthogonal sampling). The output of the 0D model comprises multiple time dependent one-dimensional signals. We focused only on quantities that are easy to measure in the clinical practice, such as systemic arterial pressure and pulmonary arterial pressure. Let us denote (0, *T*) the time interval over which the simulation of the 0D model is performed, *t*_1_ = 0, …, *t*_*n*_*t*__ = *T* a set of timesteps such that the timestep size Δ*t* = *t*_*m* + 1_ − *t*_*m*_ is constant for all *m* = 1, …, *n*_*t*_, and $u^{k,i}\left(t_{m}\right)$ the value of the *i*th quantity of interest (e.g., systemic or pulmonary arterial pressure) of the *k*th sample at timestep *t*_*m*_. In order to better capture the time component of the quantity of interest, we decided to operate on its representation in the frequency domain. More precisely, we computed the Discrete Fast Fourier Transform (DFFT) of the signal $u^{k,i}\left(t_{m}\right)$ and we recovered the coefficients $a_{j}^{k,i}$ and $b_{j}^{k,i}$ such that, for all *k* = 0, …, *n*_*t*_,

(5) $$\begin{array}{r} u^{k,i}\left(t_{m}\right)=\frac{a_{0}^{k,i}}{2}+\overset{M}{\underset{j=1}{\sum }}\left[a_{j}^{k,i}\cos\left(j\omega t_{m}\right)+b_{j}^{k,i}\sin\left(j\omega t_{m}\right)\right], \end{array}$$

where *M* = *n*_*t*_/2 and ω = 2π/*T*. We note that Equation (5) must be slightly modified to account for the case of *n*_*t*_ odd; we refer to Quarteroni et al. (2014) for details. We introduce for sake of clarity the vector ${\text{c}}^{k,i}=\left[a_{0}^{k,i},\ldots ,a_{M}^{k,i},b_{1}^{k,i},\ldots ,b_{M}^{k,i}\right]$, i.e., the vector containing all the 2*M* + 1 Fourier coefficients. In this work we selected the first 5% of Fourier coefficients, i.e., *M* = 5. Cutting higher modes allows to eliminate noise without loosing information. We studied the influence of the number of Fourier coefficients used for the DNN input on the DNN performance. Data from these studies are provided in Supplementary Data Sheet 2. Overall, results indicate that the best accuracy of the DNN is obtained using M = 5 (11 Fourier coefficients).

The training dataset *Q* for the DNN is composed of pairs {**x**^*k*^, **y**^*k*^}, *k* = 1, …, *N*_s_, where ${\text{x}}^{k}=\left[{\text{c}}^{k,1},\ldots ,{\text{c}}^{k,N_{\text{i}}}\right]$, *N*_i_ being the number of desired input signals selected from the output of the 0D model, and ${\text{y}}^{k}=\left[E_{\text{max,lv}}^{k},E_{{\text{max}}_{\text{lv},0}}^{k},G_{E_{\text{max,lv}}}^{k},k_{\text{E,lv}}^{k}\right]$. In our implementation, the input data is organized in a three-dimensional tensor $X_{lmn}=c_{n}^{l,m}$ with dimensions *N*_s_ × *N*_i_ × (2*M* + 1) as depicted in Figure 1. Therefore, the forward pass of the DNN consists in mapping the frequency coefficients of a handful of time dependent signals—in our case, systemic and pulmonary arterial pressure—to the corresponding values of the hidden parameters of the 0D model (as already mentioned in section 1, the DNN aims at solving an inverse problem). Regarding the choice of loss function $L_{\omega }\left(Q\right)$, in this work we focused on the mean squared error ${\text{MSE}}_{\omega }\left(Q\right)=1/N_{s}\overset{N_{s}}{\underset{k=1}{\sum }}|{\text{y}}^{k}-\text{f}\left({\text{x}}^{k};\omega \right){|}^{2}$, which is well-suited to regression tasks.

## 3. Results and discussion

The 0D model was implemented in the Modelica language^1^, a non-proprietary, object-oriented, equation based language which conveniently models complex physical systems. We used OpenModelica^2^ as a modeling and simulation environment. The DNN was implemented using TensorFlow^3^. Discrete fast Fourier transform computation and statistical analysis were performed with Matlab^4^.

**Figure 3:**
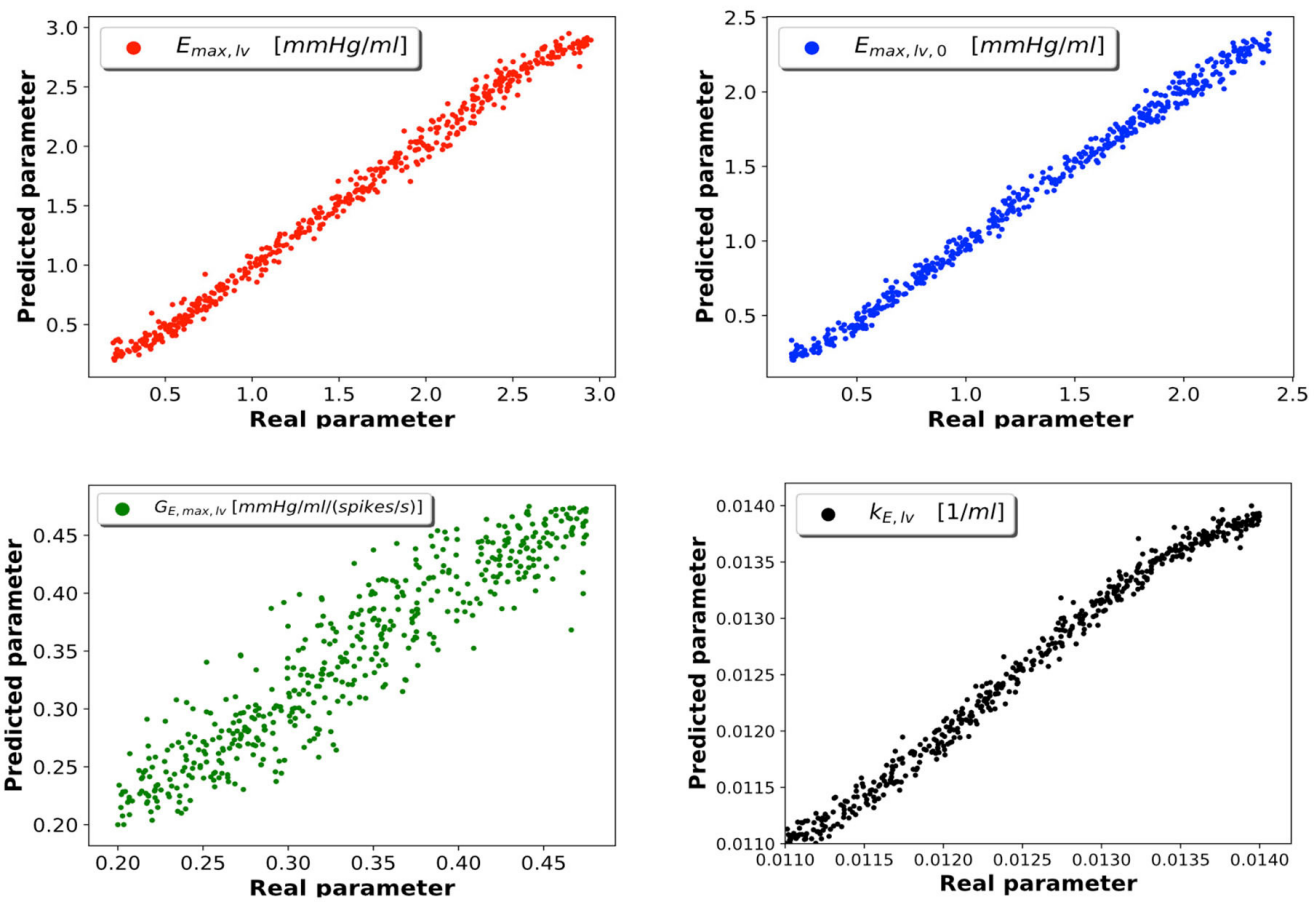
DNN performance for the 4 predicted parameters. Each plot compares the real parameter on the *x*-axis with the predicted one with the DNN on the *y*-axis. *E*_max,lv_ [mmHg/ml]: reference value of ventricle elastance, *E*_max_lv,0__ [mmHg/ml]: reference value of ventricle elastance in absence of autoregulation, *G*_*E*_max,lv__ [mmHg/ml/(spikes/s)]: maximum baroreceptor gain, *k*_E,lv_ [1/ml]: steepness of the pressure-volume curve.

As described in section 2.4, $N_{\text{s}}=10^{\prime }000$ samples were generated from the 0D model; 5% of these were reserved to the test set, which was employed to evaluate the performance of the trained network. Of the 95% samples remaining, 80% represented the train set and 20% the validation set. After considering several DNN architectures, we opted for a multi-layer perceptron with 4 fully connected hidden layers featuring 16 neurons per layer. Our choice was driven by the value of loss function achieved during training and by the absence of overfitting (which occurs whenever the error on the train set is considerably smaller than that on the validation set); in cases of architectures achieving similar performance, we chose the network composed of the smallest number of neurons. All the data regarding the considered DNN architectures and the relative performances can be found in the Supplementary Data Sheet 1. The learning rate is set at a value of 0.001. The Adam optimization algorithm was used to update the network weights during training. In Figure 3 we report the DNN performance for the 4 predicted parameters on the test set. Each graph represents the plotting of values of real parameter (*x*-axis) and the predicted parameter (*y*-axis). We note that the optimal prediction corresponds to the bisector. The architecture shows good accuracy especially for *E*_max,lv_, *E*_max_lv,0__, and *k*_E,lv_. In contrast, the prediction of *G*_*E*_max,lv__ appeared less accurate, which suggests that the 0D model is significantly less sensitive to this parameter.

To support this hypothesis, we performed a sensitivity analysis of the output of the 0D model with respect to small variations of the 4 parameters. The mean value of parameter *P* is given by *P*_*mean*_ = (*P*_*min*_ + *P*_*max*_)/2, where *P*_*min*_ and *P*_*max*_ are the lower and upper bounds depicted in Figure 2. For each parameter *P*, we run a set of 100 simulations by assigning the value *P* = *P*_*mean*_(1 + ϵ*N*(0, 1)), where ϵ = 0.01 and *N*(0, 1) is a normal distribution with 0 mean and a standard deviation equal to 1, while keeping the other 3 parameters constant and equal to their respective mean values. The results are reported in Table 2. The output of interest shows a noticeably lower standard deviation with respect to small variations of *G*_*E*_max,lv__, highlighting a lower sensibility of the 0D model for this parameter.

As depicted in Figure 1 and described in section 2.1, the main objective of our study was to recover a wealth of haemodynamic values from systemic and pulmonary arterial pressure signals. Table 1 shows the results of the 0D model simulations solved for the real and predicted parameters. Specifically, the 0D model was run for each 4-parameters set of the test set (5% of total samples, i.e., 500 samples), both with the real and the predicted parameters. Relevant haemodynamic values were extracted and compared. These values were: systemic and pulmonary pressures (mean, systolic, and diastolic), heart rate, left ventricular ejection fraction, left ventricular end-diastolic and end-systolic volumes, cardiac index, and pulmonary capillary pulmonary wedge pressure. Minimal, maximal, mean, and standard deviation of the real and predicted values were calculated. The calculated difference between real and predicted values was defined as the error, for which standard deviation with 95% confidence interval were computed. All values are reported in Table 1.

**Table 1:** Comparison between real and DNN-predicted hemodynamic data on the test set (500 samples).

	**MSAP**		**SSAP**		**DSAP**		**MPAP**		**SPAP**		**DPAP**	
	**Exact**	**Predicted**	**Exact**	**Predicted**	**Exact**	**Predicted**	**Exact**	**Predicted**	**Exact**	**Predicted**	**Exact**	**Predicted**
Min	67.924	67.870	86.812	87.348	57.464	56.761	16.320	16.349	27.687	27.746	10.637	10.650
Max	98.862	98.652	136.410	135.906	80.146	80.025	23.555	23.943	32.611	32.735	19.027	19.547
Mean	88.158	88.042	120.373	120.158	72.051	71.984	18.890	18.907	29.545	29.563	13.562	13.579
SD	6.245	6.549	10.241	10.678	4.347	4.593	1.351	1.427	0.892	0.945	1.583	1.672
Mean Err	0.499		0.841		0.357		0.114		0.099		0.129	
Mean Rel Err	0.006		0.007		0.005		0.006		0.003		0.009	
Err SD	0.484		0.810		0.346		0.106		0.083		0.127	
Err CI low	0.456		0.770		0.326		0.104		0.091		0.118	
Err CI up	0.541		0.912		0.387		0.123		0.106		0.140	
	**Heart rate**		**LVEF**		**LVEDV**		**LVESV**		**CI**		**PCWP**	
	**Exact**	**Predicted**	**Exact**	**Predicted**	**Exact**	**Predicted**	**Exact**	**Predicted**	**Exact**	**Predicted**	**Exact**	**Predicted**
Min	60.000	60.000	29.542	28.523	138.541	139.436	56.173	55.597	1.662	1.653	6.251	6.273
Max	78.947	83.333	61.178	60.764	217.007	220.059	152.898	157.291	3.084	3.087	15.863	16.113
Mean	65.731	65.836	49.613	49.676	162.961	162.828	83.056	82.943	2.621	2.617	9.712	9.744
SD	4.084	4.426	7.032	7.295	15.486	15.963	19.241	19.912	0.308	0.316	1.868	1.970
Mean Err	0.903		0.493		1.138		1.342		0.035		0.152	
Mean Rel Err	0.014		0.011		0.007		0.015		0.014		0.015	
Err SD	1.376		0.433		1.028		1.214		0.031		0.138	
Err CI low	0.783		0.455		1.048		1.235		0.032		0.140	
Err CI up	1.024		0.531		1.229		1.448		0.038		0.164	

*For each haemodynamic variable, minimal, maximal, mean, and standard deviation values are given for the exact and predicted samples. For each set of data in the 500 samples, the difference between exact and predicted values (error) was calculated, and mean error (absolute and relative ) was computed, with additional calculation of its standard deviation and its 95% confidence interval. MSAP [mmHg], mean systemic arterial pressure; SSAP [mmHg], systolic systemic arterial pressure; DSAP [mmHg], diastolic systemic arterial pressure; MPAP [mmHg], mean pulmonary arterial pressure; SPAP [mmHg], systolic pulmonary arterial pressure; DMAP [mmHg], diastolic pulmonary arterial pressure; LVEF [%], left ventricle ejection fraction; LVEDV [ml], left ventricle end-diastolic volume; LVESV [ml], left ventricle end-systolic volume; CI [L/(min m^2^)], cardiac index; PCWP [mmHg], pulmonary capillary wedge pressure; Min, minimal value; Max, maximal value; Mean, mean value; SD, standard deviation; Mean Err, mean error; Mean Rel Err, mean relative error; Err SD, error standard deviation; Err CI min, error confidence interval lower bound; Err CI up, error confidence interval upper bound*.

**Table 2:** Sensitivity analysis for the 4 predicted parameters, performed as described in section 3.

		**MSAP**	**SSAP**	**DSAP**	**MPAP**	**SPAP**	**DPAP**	**HR**	**LVEF**	**LVEDV**	**LVESV**	**CI**	**PCWP**
***E*_max,lv_**													
	Min	88.112	121.471	70.979	17.733	28.827	12.186	62.500	44.071	155.524	69.532	2.491	8.469
	Max	90.846	123.547	74.516	19.762	30.133	14.576	68.182	55.292	167.613	93.507	2.820	10.487
	Mean	89.400	122.586	72.807	18.697	29.415	13.338	64.746	49.676	161.103	81.187	2.671	9.381
	SD	0.737	0.542	0.908	0.600	0.346	0.729	1.504	3.398	3.385	7.179	0.120	0.578
***E*_max_lv,0__**													
	Min	76.391	99.962	64.606	17.582	28.621	11.986	60.000	39.089	146.217	61.539	2.011	7.706
	Max	95.113	132.031	76.921	20.514	30.713	15.415	75.000	57.912	187.664	114.309	3.017	12.551
	Mean	88.207	120.344	72.138	18.759	29.451	13.413	65.651	49.809	162.815	82.347	2.637	9.557
	SD	5.350	9.204	3.428	0.865	0.564	1.018	3.763	5.185	12.310	14.920	0.308	1.385
***G*_*E*_max,lv__**													
	Min	88.120	119.649	72.355	18.213	29.165	12.727	62.500	48.984	157.368	76.024	2.508	8.897
	Max	90.513	124.818	73.361	18.927	29.537	13.651	68.182	51.690	163.877	83.603	2.847	9.649
	Mean	89.416	122.375	72.936	18.672	29.354	13.331	64.523	50.382	160.505	79.654	2.717	9.257
	SD	0.580	1.310	0.261	0.164	0.099	0.200	1.506	0.778	1.874	2.178	0.094	0.214
***k*_E,lv_**													
	Min	86.914	118.793	70.946	17.543	28.569	11.974	62.500	50.196	152.245	75.199	2.413	7.995
	Max	92.191	126.766	74.940	19.559	30.043	14.317	68.182	50.614	168.665	84.003	2.955	10.455
	Mean	89.583	122.755	72.998	18.537	29.309	13.151	64.723	50.428	160.677	79.657	2.708	9.190
	SD	1.583	2.488	1.141	0.650	0.425	0.764	2.124	0.133	5.080	2.730	0.187	0.7583

*Minimal, maximal, mean and standard deviation are reported for each haemodynamic variable. MSAP [mmHg], mean systemic arterial pressure; SSAP [mmHg], systolic systemic arterial pressure; DSAP [mmHg], diastolic systemic arterial pressure; MPAP [mmHg], mean pulmonary arterial pressure; SPAP [mmHg], systolic pulmonary arterial pressure; DMAP [mmHg], diastolic pulmonary arterial pressure; LVEF [%], left ventricle ejection fraction; LVEDV [ml], left ventricle end-diastolic volume; LVESV [ml], left ventricle end-systolic volume; CI [L/(min m^2^)], cardiac index; PCWP [mmHg], pulmonary capillary wedge pressure; Min, minimal value; Max, maximal value; Mean, mean value; SD, standard deviation*.

Our test set included haemodynamic values spanning a range of clinical situation from normal physiology to severe heart failure. Overall our results indicate an excellent correlation between real and predicted values, with mean relative error never superior to 2%, and a narrow 95%-confidence interval. The accuracy of this model is relevant to the clinical setting, where these hemodynamic variables are susceptible to show wide variations.

Furthermore, the small error in our model should be regarded as perfectly acceptable, when considering the variability of intravascular pressure measurements in the clinical setting, notably related to underdamping and resonance phenomena (Romagnoli et al., 2014). The same is true for clinical measurements of ventricular volumes (Bastos et al., 2019). With respect to ventricular ejection fraction, it is worth mentioning that its measurement by standard modern techniques (cardiac computerized tomography, radionucleotide and invasive ventriculography, echocardiography or magnetic resonance imaging) presents a bias of less than 5% (Pickett et al., 2015), which reinforces the relevance of our model, which leads to a relative error smaller than 2%. Finally, new devices and non-invasive approaches for continuous pressure measurement may broaden the applicability of the presented framework (Proena et al., 2016), while new methods for haemodynamic monitoring could provide innovative perspectives (Pour-Ghaz et al., 2019).

Our model appears slightly better to predict pressures than other parameters, which may reflect the fact that the DNN takes pressure, but not volumes values, as input. In addition, with respect of heart rate, it can be noticed that minimal predicted and real values were identical (60/min), which simply reflects the lowest boundary of this parameter in the 0D model.

## 4. Limitations

We acknowledge the three following limitations in our study. First, our model is adapted to the pathophysiology of left heart failure, and therefore cannot apply to other condition of cardiac failure, such as right heart dysfunction. Secondly, we choose to focus only on 4 parameters of interest. Therefore, it is likely that including additional parameters (for instance a resistance parameter associated with vascular dysfunction) could have affected the resulting accuracy of the network. Third, even though the 0D model includes a component of autoregulation, it does not take into account respiratory physiological variables, which may significantly influence cardiac function (concept of heart-lung interactions), notably in the context of mechanical ventilation, which is frequently provided in patients with severe cardiac failure. Fourth, our study used exclusively synthetic data. Using real patients data (possibly affected by noise) to validate our framework will be essential for its applicability in the clinical setting, and such validation will be the matter of future investigations. Furthermore, we cannot rule out that using real clinical data could affect the ability to train the proposed network, an issue that will require additional studies.

## 5. Conclusions and Perspectives

In this work we presented a framework that allows to predict, from physiological data (systemic and pulmonary arterial pressure signals), various parameters of left ventricular function and other haemodynamic variables. The 0D model used to train the DNN in our study allows to describe a wide spectrum of pathophysiological alterations pertaining to left heart failure. In turn, the DNN displayed remarkable accuracy to recover relevant haemodynamic parameters.

Our study represents a first step toward the development of automated tools providing helpful information on heart function. For instance, the application of our framework could assist in the real time detection of left ventricular dysfunction, especially in ICU patients, who are subject to continuous monitoring of intravascular pressures. Also, our tool could be useful to assess the left ventricular function during mechanical circulatory support, such as left ventricular assist device (LVAD) or veno-arterial extracorporeal membrane oxygenation (ECMO).

Future studies, that will validate the model with patient related data, will be necessary for the clinical implementation of our tool. Moreover, our framework will be of great interest for the future extensions of the 0D model to other circulatory pathologies or heart disease as well as to mechanical circulatory support.

## Data Availability Statement

The raw data supporting the conclusions of this article will be made available by the authors, without undue reservation. Implementation of the lumped parameter model can be found at: https://bitbucket.org/jbonnema/lumped_parameter_model/src/master/.

## Author Contributions

JB contributed to the design and the implementation of the research, to the analysis of results and to the writing of the manuscript. LP contributed to the design and the implementation of the research, and to the writing of the manuscript. SD contributed to the design of the research, to the analysis of the results, and to the writing of the manuscript. LL contributed to the analysis of the research and to the writing of the manuscript. All authors contributed to the article and approved the submitted version.

## Conflict of Interest

The authors declare that the research was conducted in the absence of any commercial or financial relationships that could be construed as a potential conflict of interest.

## Footnotes

^1^http://www.modelica.org

^2^http://www.openmodelica.org

^3^https://www.tensorflow.org

^4^https://www.mathworks.com
