# Supplementary material for: Implementation and Calibration of a Deep Neural Network to Predict Parameters of Left Ventricular Systolic Function Based on Pulmonary and Systemic Arterial Pressure Signals
Source: Front Physiol. 2020 Sep 11;11:1086. doi: 10.3389/fphys.2020.01086 (PMC7533610; doi:10.3389/fphys.2020.01086)

Supplementary materials

DNN architecture evaluation

- *"Implementation and calibration of a deep neural network to predict parameters of left ventricular systolic function based on pulmonary and systemic arterial pressure signals"*
- Jean Bonnemain, Luca Pegolotti, Lucas Liaudet, Simone Deparis
- Brief Research Report, Front. Physiol. - Computational Physiology and Medicine

DNN architecture evaluation

- Page 3 contains a table with all the tested architectures and results in terms of mean absolute error and loss.
- The selected architecture for this work is in red, i.e., architecture number 6.
- Page 4 and following show in detail for each architecture the results, that contain:
  - Description of the architecture
  - A plot of real parameter against predicted parameter.
  - A plot of loss and validation loss against epoch.
  - Errors and losses

- Abbreviations
  - MSE : Mean Squared Error
  - MAE : Mean Absolute Error

Parameters \ Architecture	0	1	2	3	4	5	6	7	8	9	10	11	12	13
Hidden layers	5	4	3	2	1	5	4	3	2	1	6	3	6	3
Hidden layers – activation function	Relu	Relu	Relu	Relu	Relu	Relu	Relu	Relu	Relu	Relu	Relu	Relu	Relu	Relu
Neurons	32	32	32	32	32	16	16	16	16	16	128	128	64	64
Output layer – activation function	Sigmoid	Sigmoid	Sigmoid	Sigmoid	Sigmoid	Sigmoid	Sigmoid	Sigmoid	Sigmoid	Sigmoid	Sigmoid	Sigmoid	Sigmoid	Sigmoid
Optimizer	Adam	Adam	Adam	Adam	Adam	Adam	Adam	Adam	Adam	Adam	Adam	Adam	Adam	Adam
Loss	MSE	MSE	MSE	MSE	MSE	MSE	MSE	MSE	MSE	MSE	MSE	MSE	MSE	MSE
Metrics	MAE	MAE	MAE	MAE	MAE	MAE	MAE	MAE	MAE	MAE	MAE	MAE	MAE	MAE
Epochs	1000	1000	1000	1000	1000	1000	1000	1000	1000	1000	1000	1000	1000	1000
Loss	0.0021	0.0023	0.0024	0.0027	0.0041	0.0028	0.0027	0.0031	0.0035	0.0045	0.0019	0.0012	0.0012	0.0017
Validation loss	0.0021	0.0023	0.0023	0.0027	0.0041	0.0026	0.0028	0.0029	0.0034	0.0045	0.0024	0.0019	0.0016	0.0021
Mae	0.0307	0.0317	0.0322	0.0355	0.0457	0.0362	0.0357	0.0385	0.0412	0.0491	0.0300	0.0231	0.0243	0.0273
Validation mae	0.0304	0.0317	0.0314	0.0354	0.0456	0.0347	0.0364	0.0369	0.0408	0.0487	0.0317	0.0273	0.0263	0.0322

Architecture 0

Parameters	DNN values
Hidden layers	5
Hidden layers – activation function	Relu
Neurons	32
Output layer – activation function	Sigmoid
Optimizer	Adam
Loss	MSE
Metrics	MAE
Epochs	1000
loss: 0.0021 - mae: 0.0307 - val_loss: 0.0021 - val_mae: 0.0304	

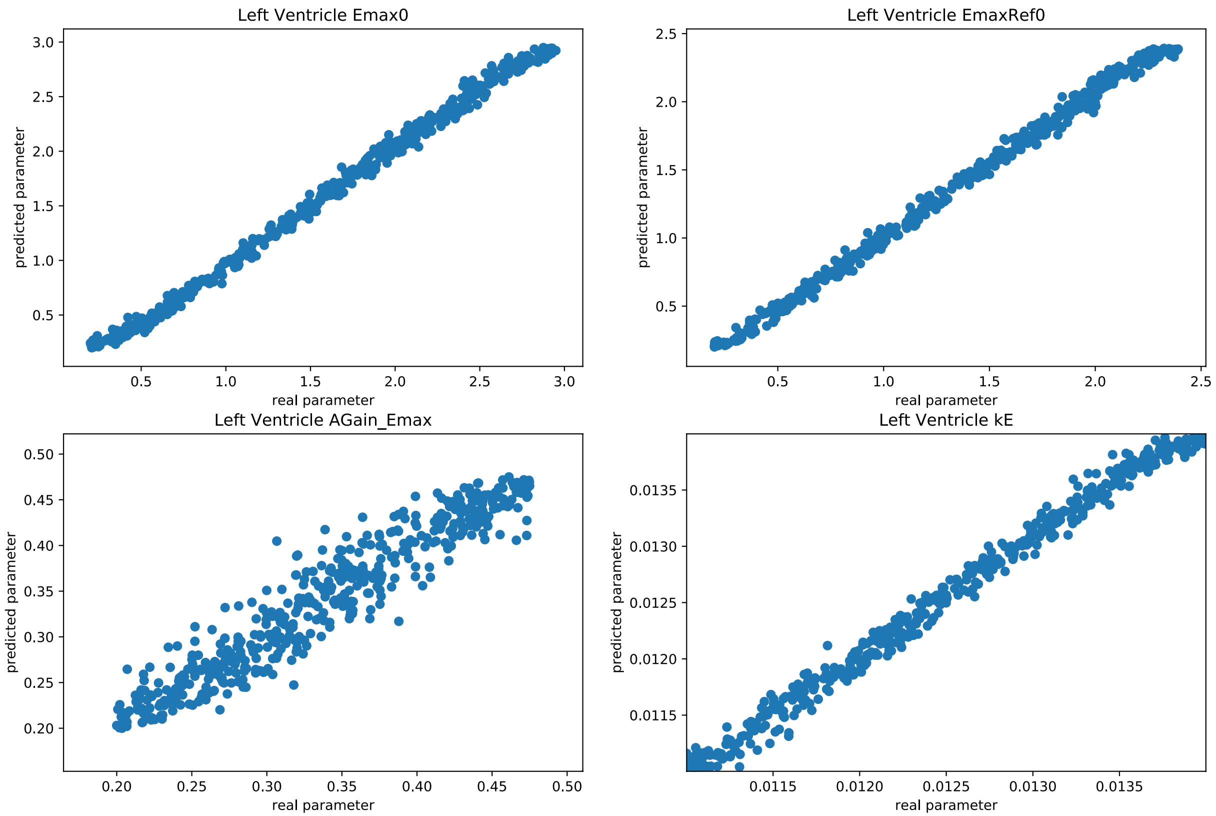

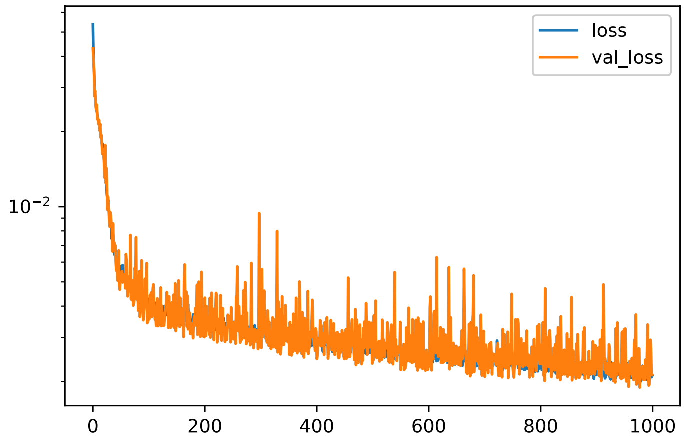

Architecture 1

Parameters	DNN values
Hidden layers	4
Hidden layers – activation function	Relu
Neurons	32
Output layer – activation function	Sigmoid
Optimizer	Adam
Loss	MSE
Metrics	MAE
Epochs	1000
loss: 0.0023 - mae: 0.0317 - val_loss: 0.0023 - val_mae: 0.0317	

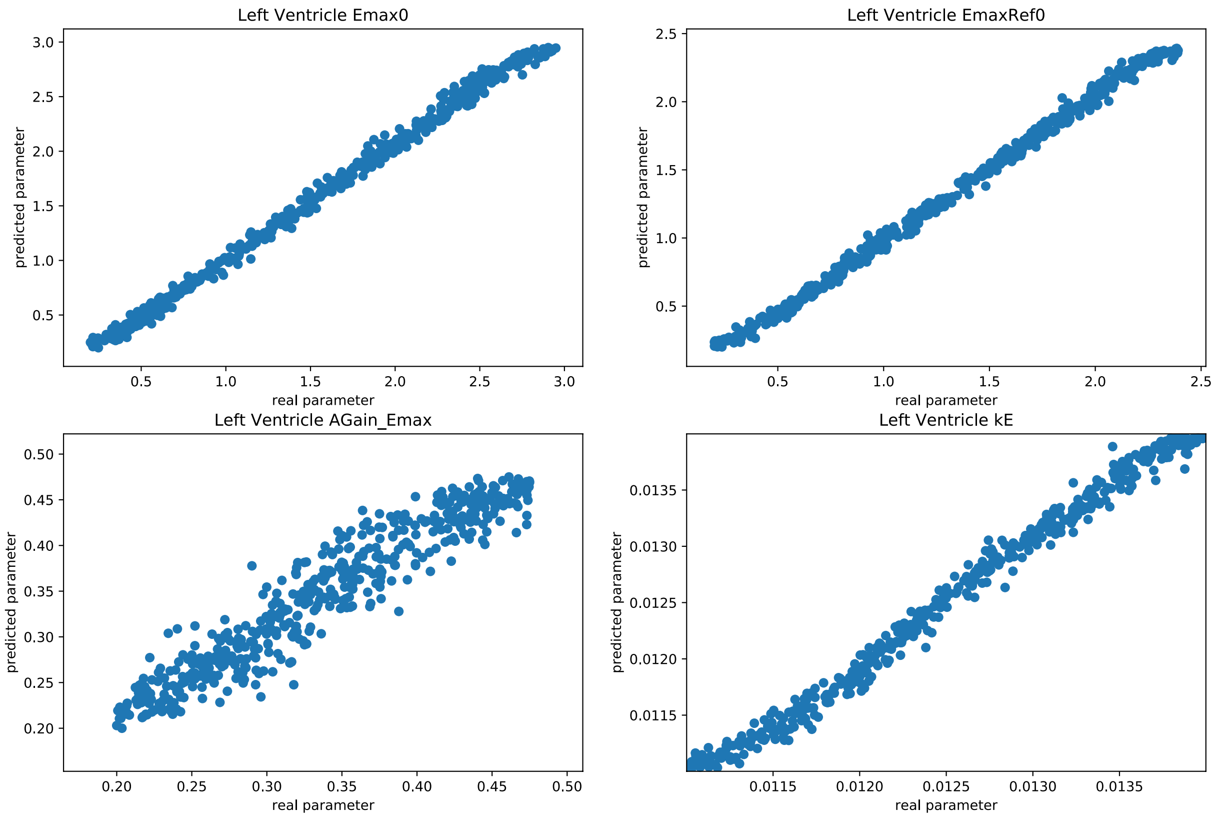

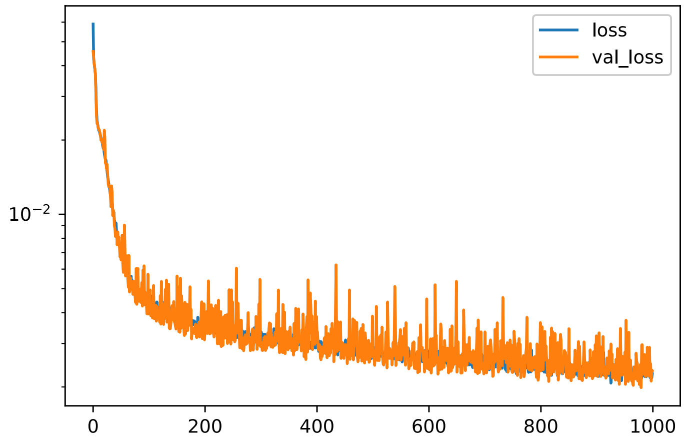

Architecture 2

Parameters	DNN values
Hidden layers	3
Hidden layers – activation function	Relu
Neurons	32
Output layer – activation function	Sigmoid
Optimizer	Adam
Loss	MSE
Metrics	MAE
Epochs	1000
loss: 0.0024 - mae: 0.0322 - val_loss: 0.0023 - val_mae: 0.0314	

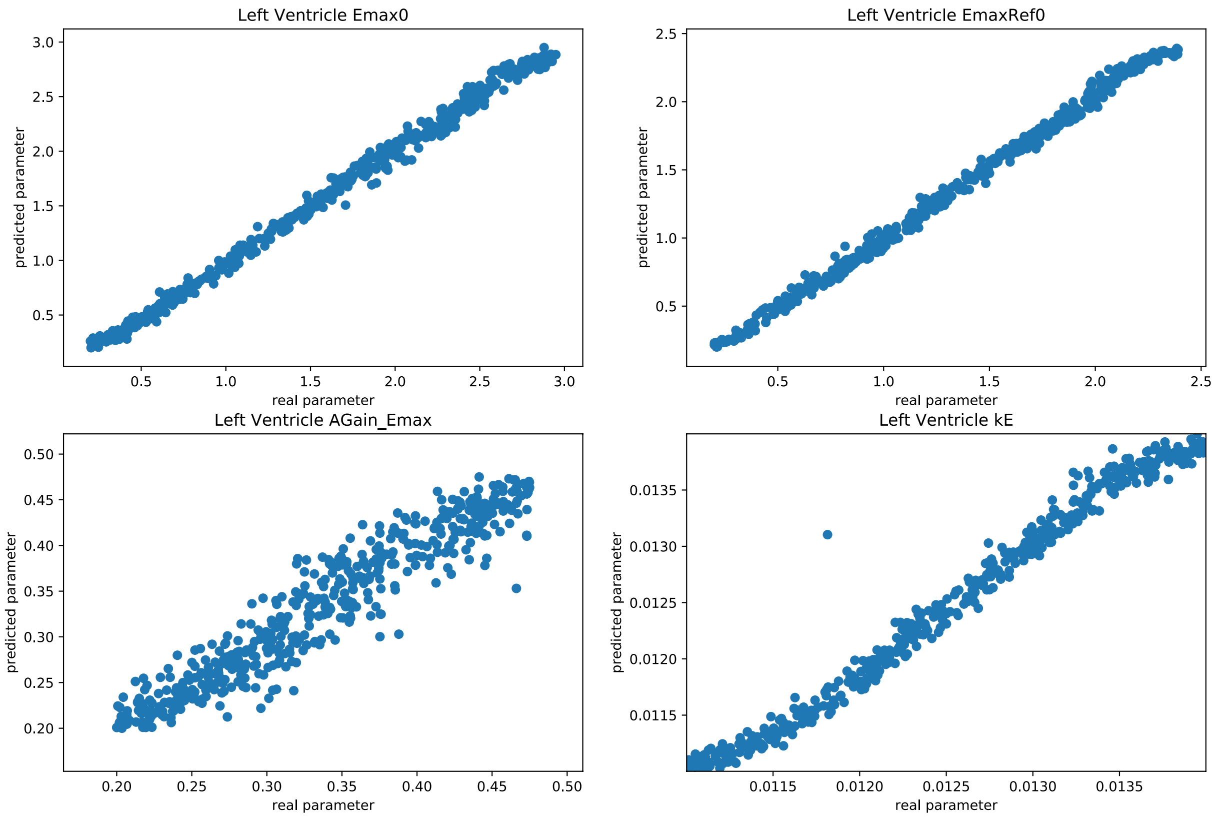

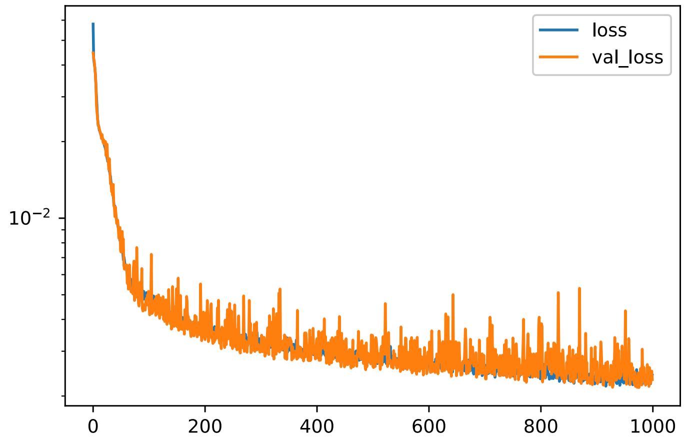

Architecture 3

Parameters	DNN values
Hidden layers	2
Hidden layers – activation function	Relu
Neurons	32
Output layer – activation function	Sigmoid
Optimizer	Adam
Loss	MSE
Metrics	MAE
Epochs	1000
loss: 0.0027 - mae: 0.0355 - val_loss: 0.0027 - val_mae: 0.0354	

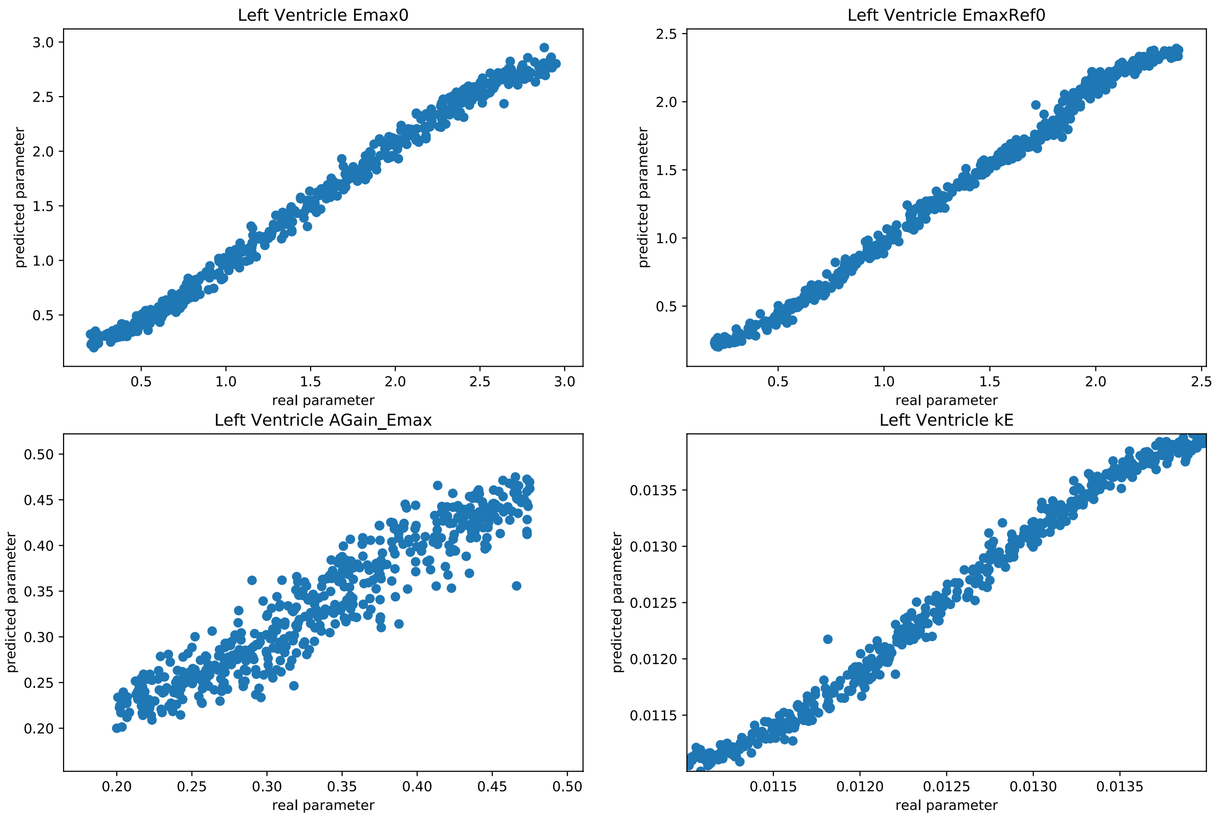

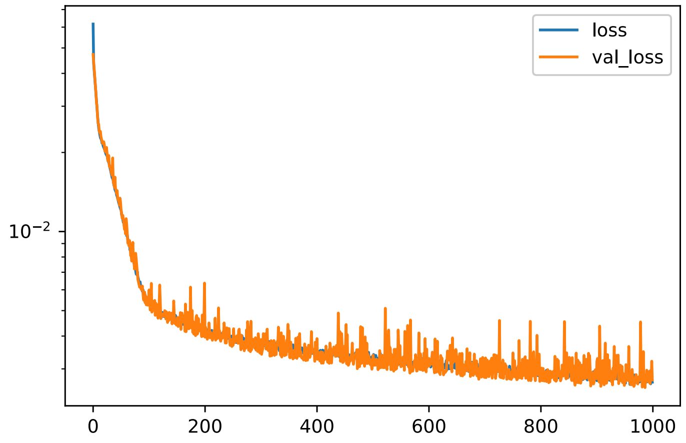

Architecture 4

Parameters	DNN values
Hidden layers	1
Hidden layers – activation function	Relu
Neurons	32
Output layer – activation function	Sigmoid
Optimizer	Adam
Loss	MSE
Metrics	MAE
Epochs	1000
loss: 0.0041 - mae: 0.0457 - val_loss: 0.0041 - val_mae: 0.0456	

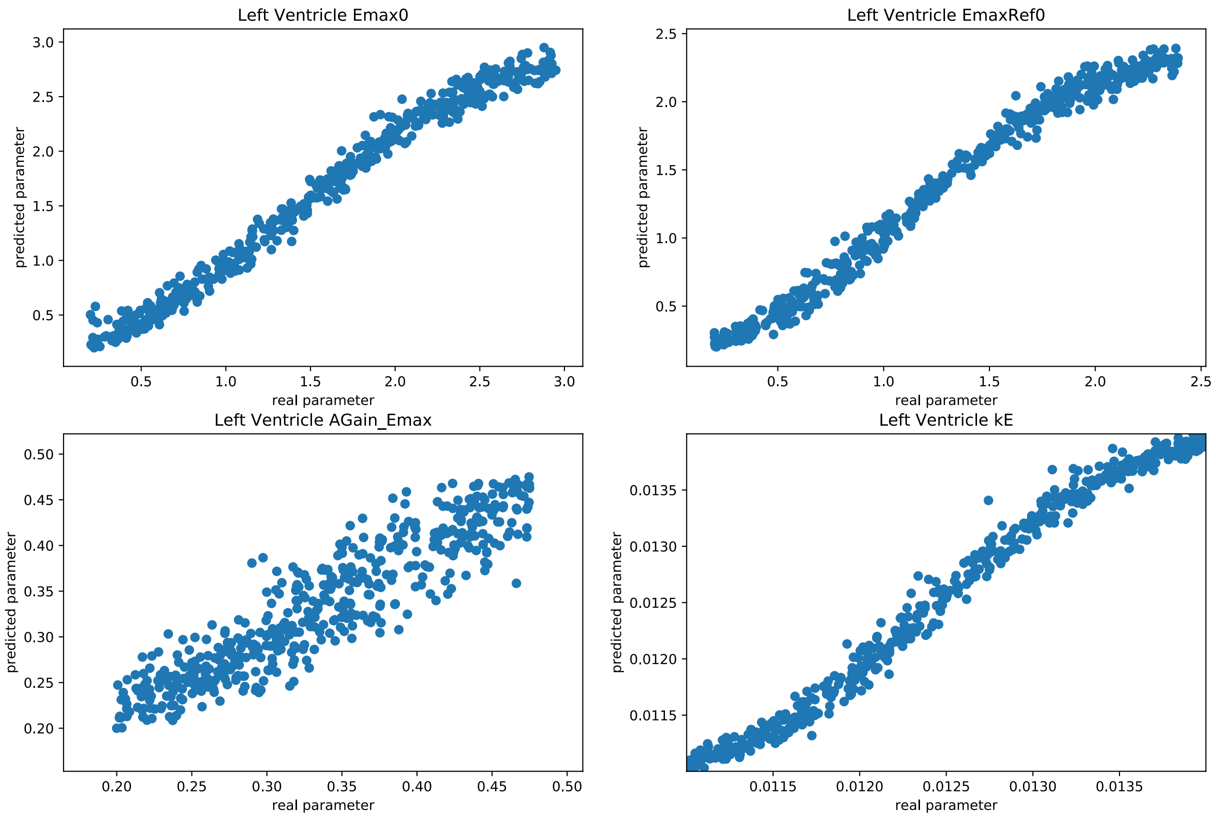

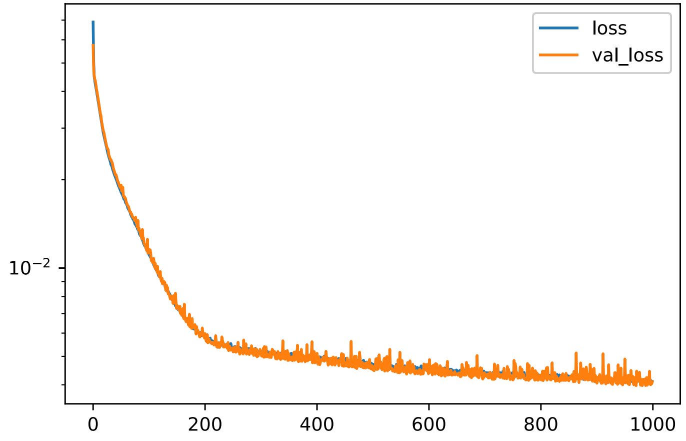

Architecture 5

Parameters	DNN values
Hidden layers	5
Hidden layers – activation function	Relu
Neurons	16
Output layer – activation function	Sigmoid
Optimizer	Adam
Loss	MSE
Metrics	MAE
Epochs	1000
loss: 0.0028 - mae: 0.0362 - val_loss: 0.0026 - val_mae: 0.0347	

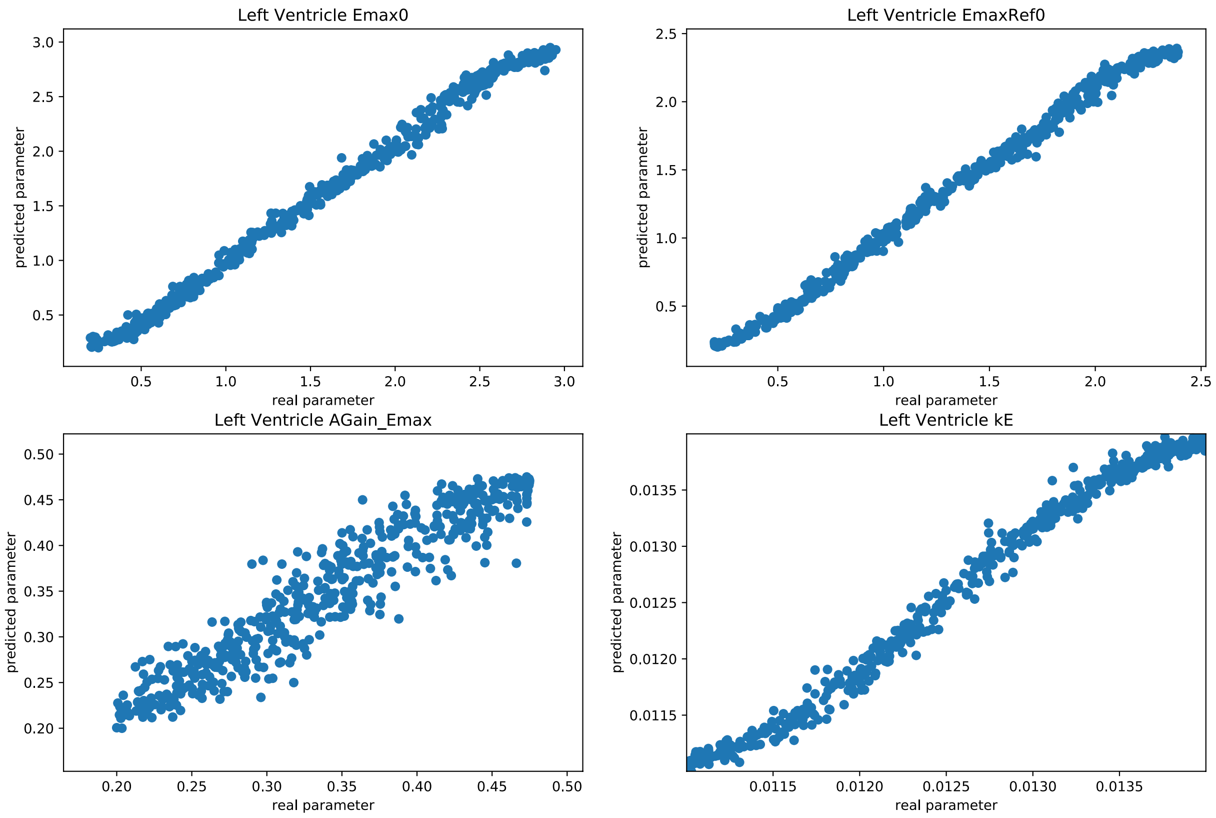

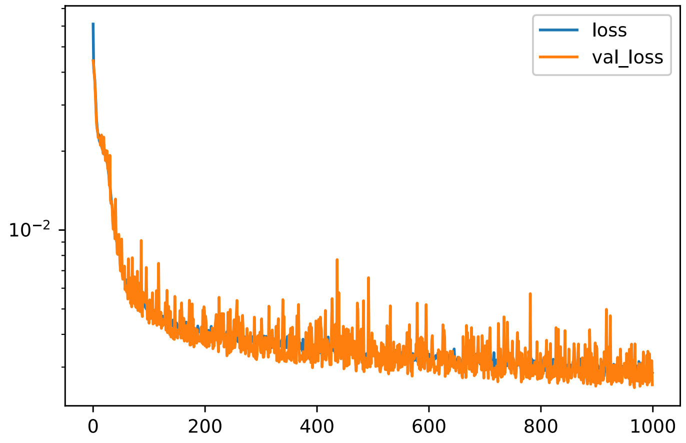

Architecture 6

Parameters	DNN values
Hidden layers	4
Hidden layers – activation function	Relu
Neurons	16
Output layer – activation function	Sigmoid
Optimizer	Adam
Loss	MSE
Metrics	MAE
Epochs	1000
loss: 0.0027 - mae: 0.0357 - val_loss: 0.0028 - val_mae: 0.0364	

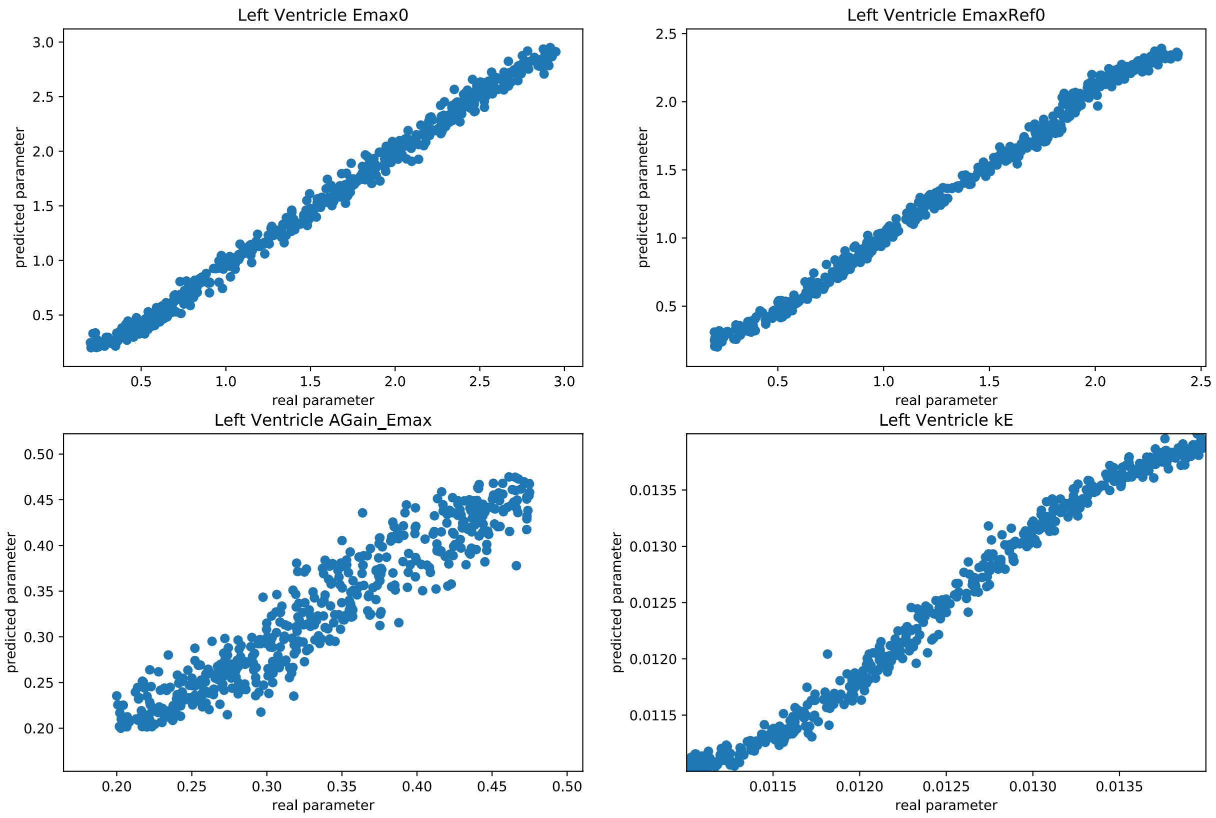

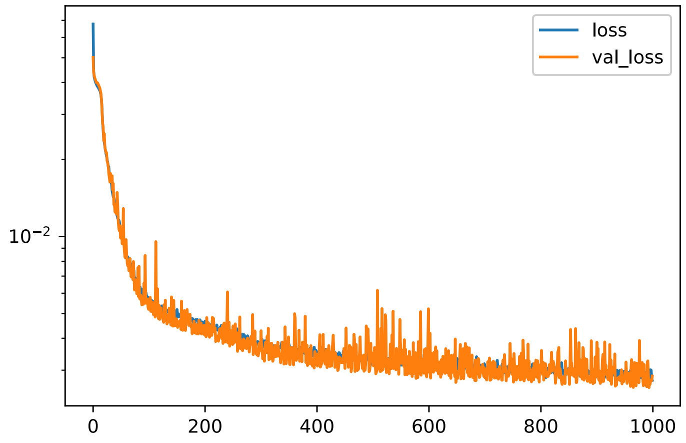

Architecture 7

Parameters	DNN values
Hidden layers	3
Hidden layers – activation function	Relu
Neurons	16
Output layer – activation function	Sigmoid
Optimizer	Adam
Loss	MSE
Metrics	MAE
Epochs	1000
loss: 0.0031 - mae: 0.0385 - val_loss: 0.0029 - val_mae: 0.0369	

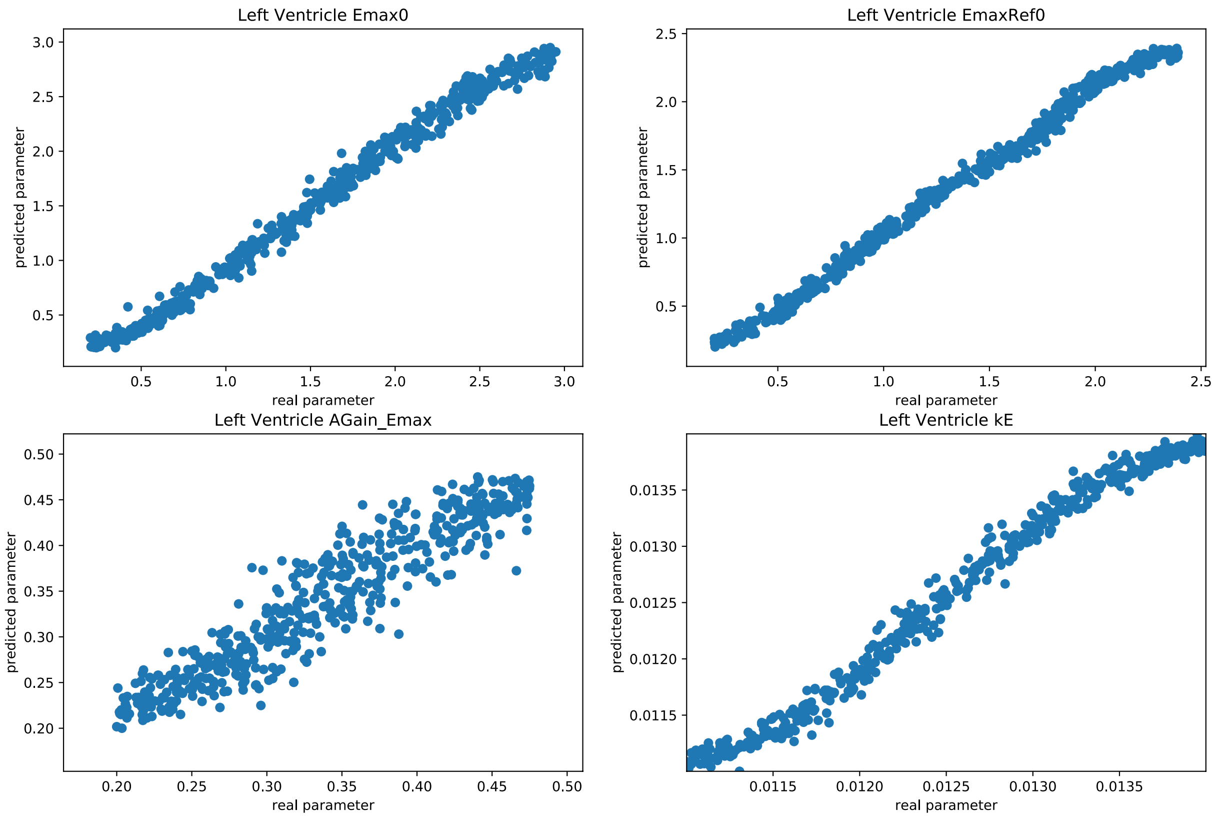

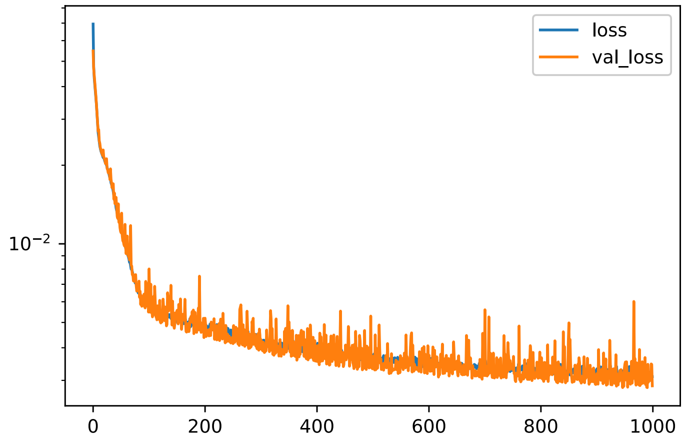

Architecture 8

Parameters	DNN values
Hidden layers	2
Hidden layers – activation function	Relu
Neurons	16
Output layer – activation function	Sigmoid
Optimizer	Adam
Loss	MSE
Metrics	MAE
Epochs	1000
loss: 0.0035 - mae: 0.0412 - val_loss: 0.0034 - val_mae: 0.0408	

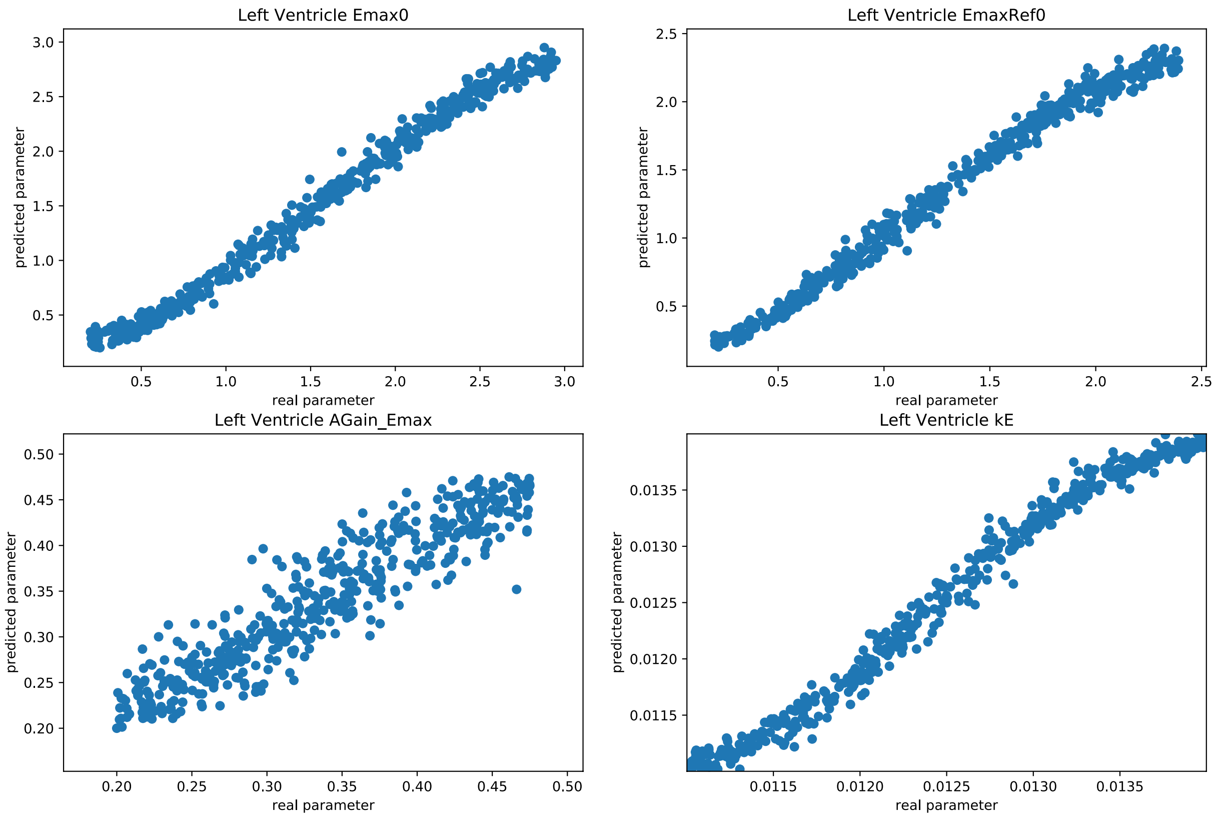

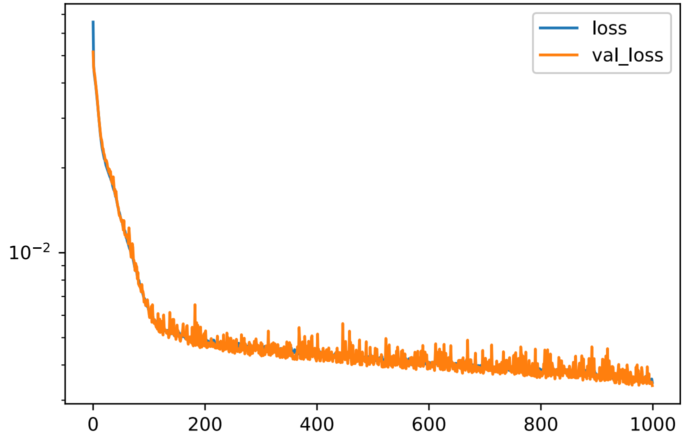

Architecture 9

Parameters	DNN values
Hidden layers	1
Hidden layers – activation function	Relu
Neurons	16
Output layer – activation function	Sigmoid
Optimizer	Adam
Loss	MSE
Metrics	MAE
Epochs	1000
loss: 0.0045 - mae: 0.0491 - val_loss: 0.0045 - val_mae: 0.0487	

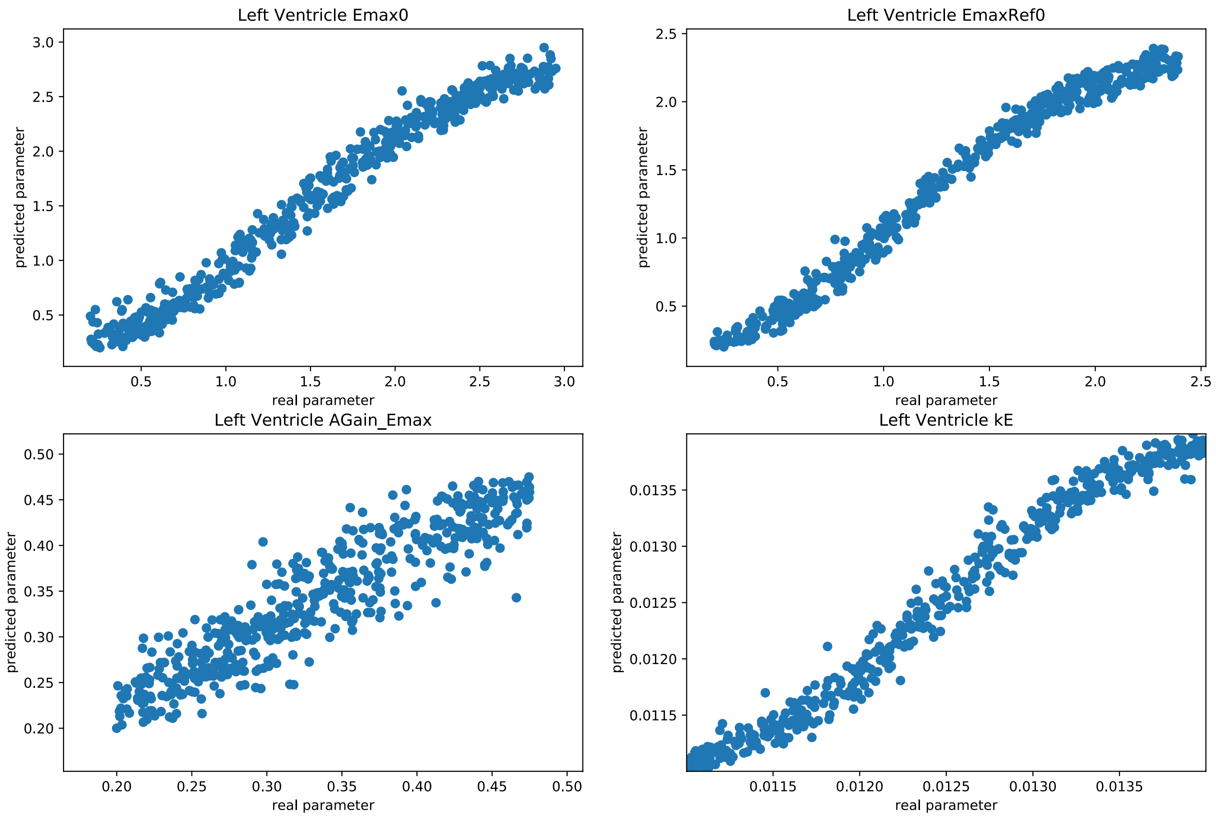

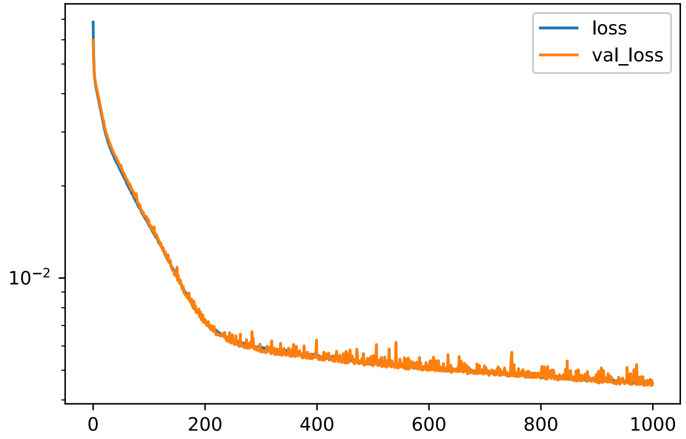

Architecture 10

Parameters	DNN values
Hidden layers	6
Hidden layers – activation function	Relu
Neurons	128
Output layer – activation function	Sigmoid
Optimizer	Adam
Loss	MSE
Metrics	MAE
Epochs	1000
loss: 0.0019 - mae: 0.0300 - val_loss: 0.0024 - val_mae: 0.0317	

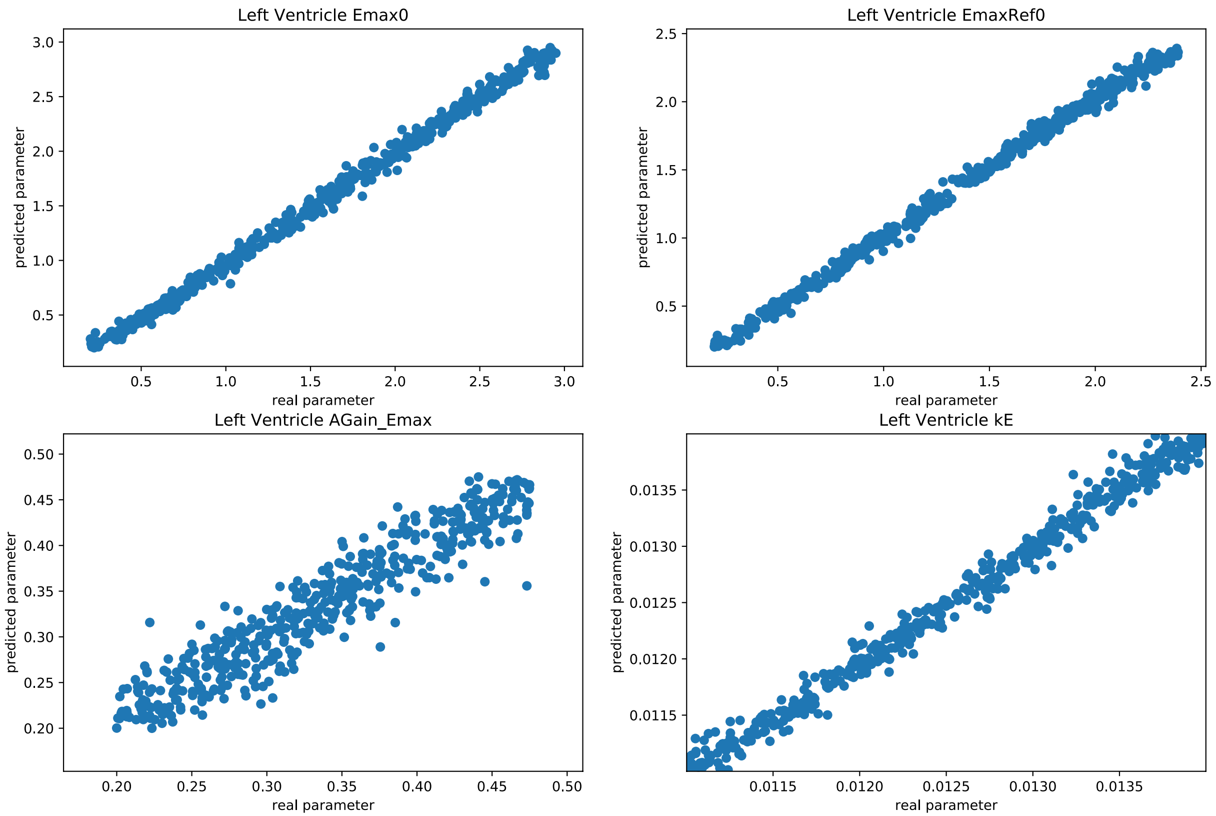

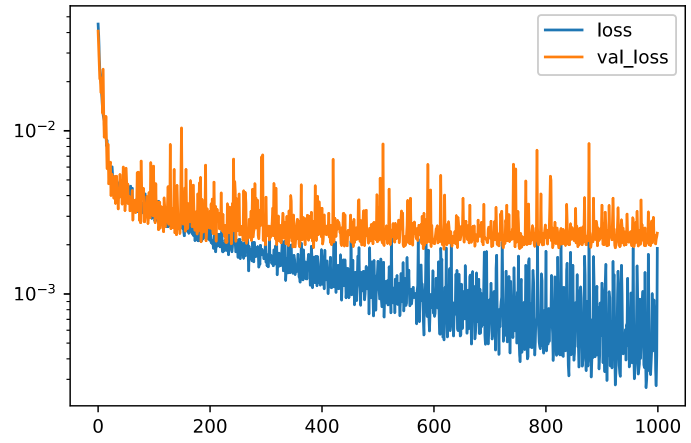

Architecture 11

Parameters	DNN values
Hidden layers	3
Hidden layers – activation function	Relu
Neurons	128
Output layer – activation function	Sigmoid
Optimizer	Adam
Loss	MSE
Metrics	MAE
Epochs	1000
loss: 0.0012 - mae: 0.0231 - val_loss: 0.0019 - val_mae: 0.0273	

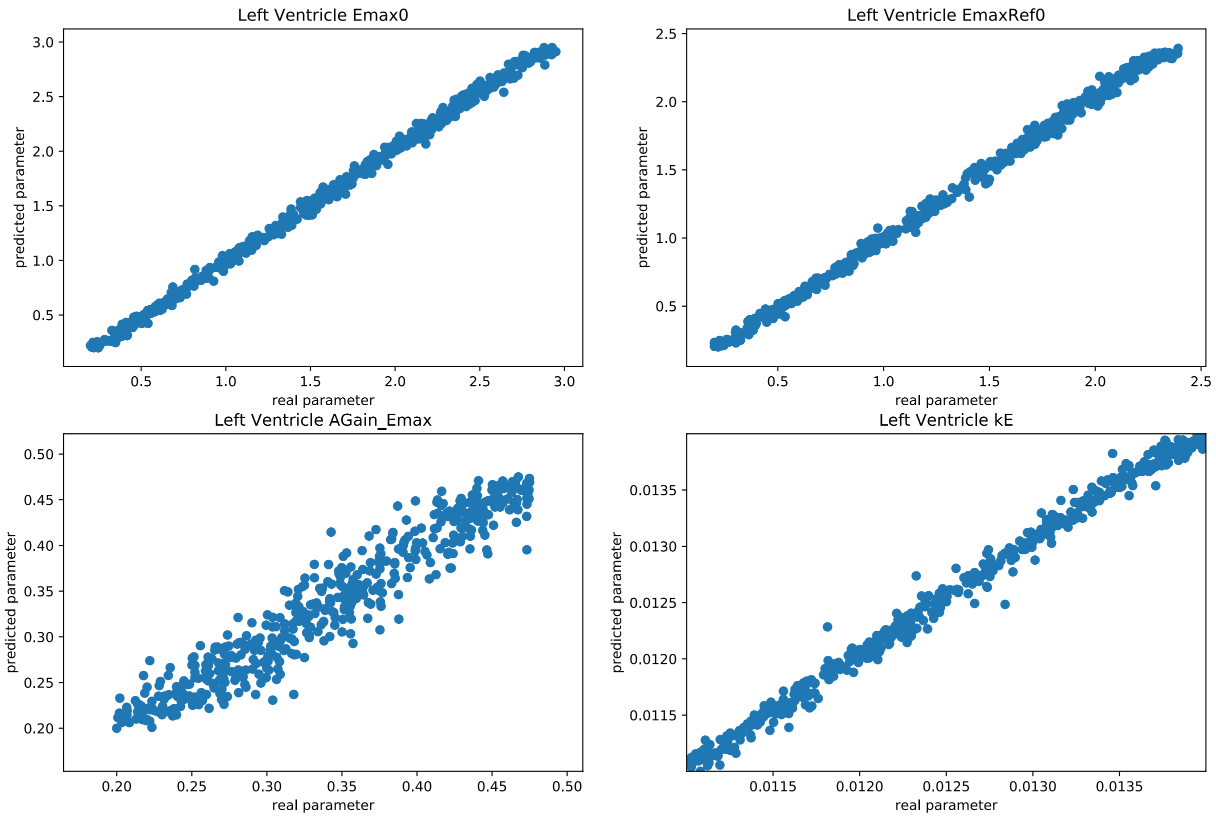

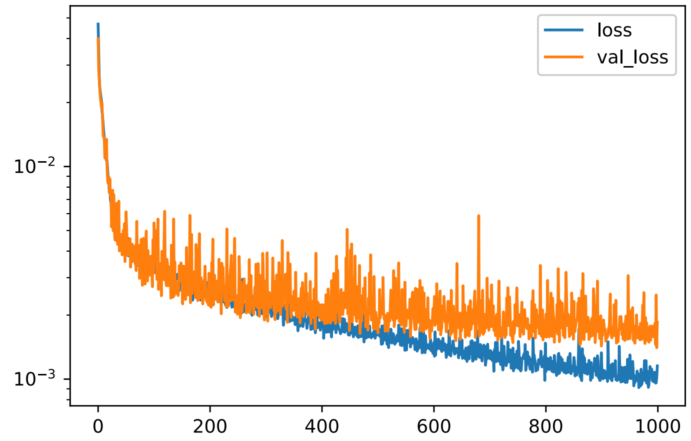

Architecture 12

Parameters	DNN values
Hidden layers	6
Hidden layers – activation function	Relu
Neurons	64
Output layer – activation function	Sigmoid
Optimizer	Adam
Loss	MSE
Metrics	MAE
Epochs	1000
loss: 0.0012 - mae: 0.0243 - val_loss: 0.0016 - val_mae: 0.0263	

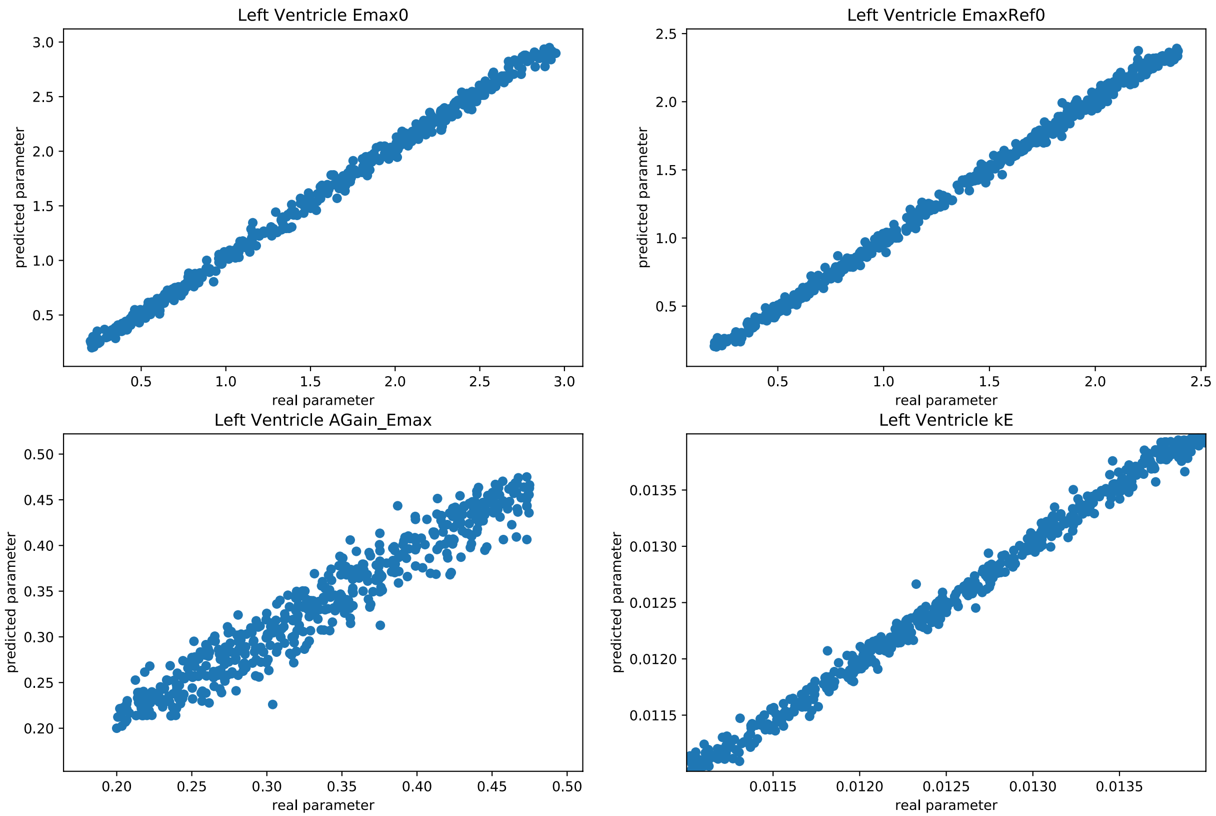

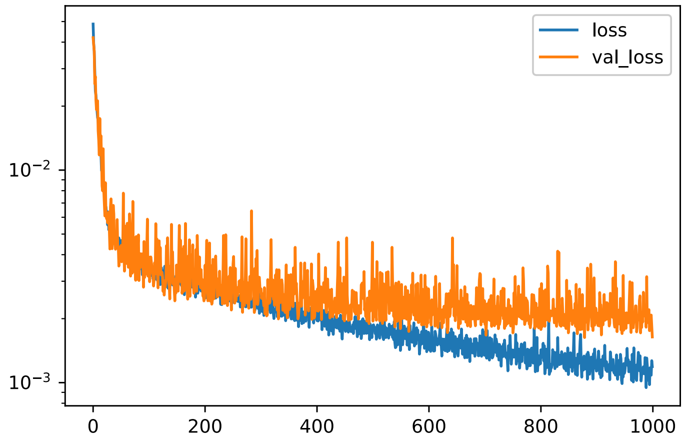

Architecture 13

Parameters	DNN values
Hidden layers	3
Hidden layers – activation function	Relu
Neurons	64
Output layer – activation function	Sigmoid
Optimizer	Adam
Loss	MSE
Metrics	MAE
Epochs	1000
loss: 0.0017 - mae: 0.0273 - val_loss: 0.0021 - val_mae: 0.0322	

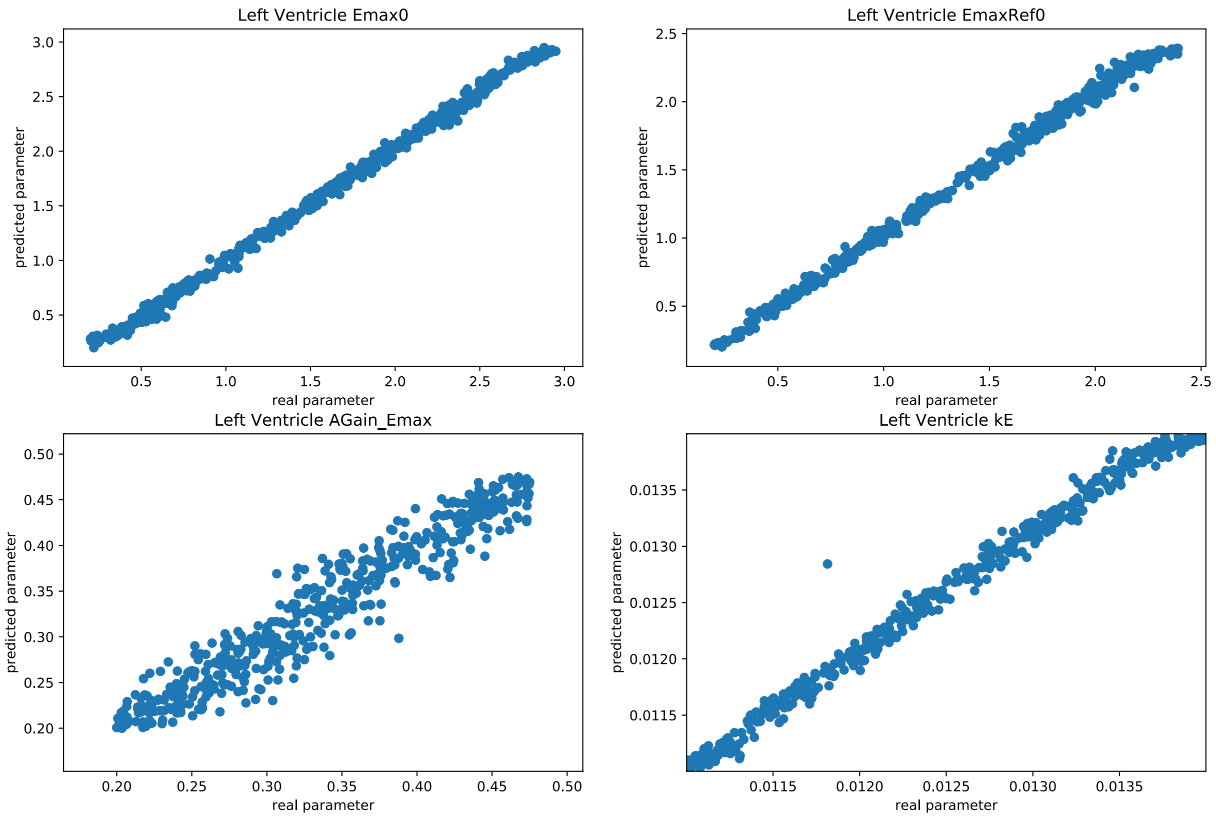

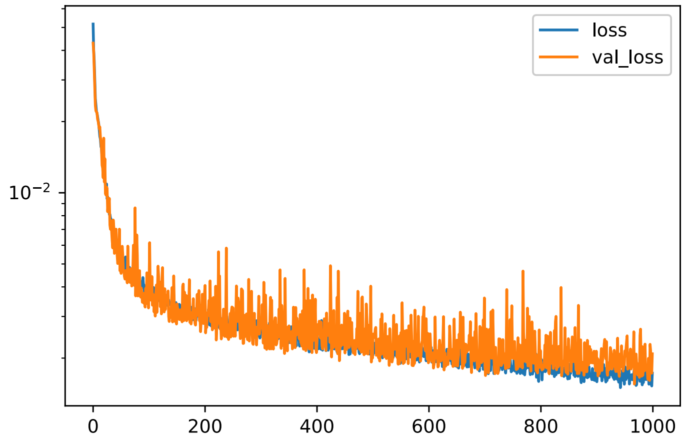

Supplement: Supplementary file 1 [file Data_Sheet_1.PDF]
