# Supplementary material for: Implementation and Calibration of a Deep Neural Network to Predict Parameters of Left Ventricular Systolic Function Based on Pulmonary and Systemic Arterial Pressure Signals
Source: Front Physiol. 2020 Sep 11;11:1086. doi: 10.3389/fphys.2020.01086 (PMC7533610; doi:10.3389/fphys.2020.01086)
Supplement: Supplementary file 2 [file Data_Sheet_2.PDF]

Supplementary materials

Fourier coefficients selection

- *"Implementation and calibration of a deep neural network to predict parameters of left ventricular systolic function based on pulmonary and systemic arterial pressure signals"*
- Jean Bonnemain, Luca Pegolotti, Lucas Liaudet, Simone Deparis
- Brief Research Report, Front. Physiol. - Computational Physiology and Medicine

Fourier coefficient selection

- Page 3 contains a table that reports the effect of selected number of Fourier coefficient for the input on the DNN performance.
- All tests are performed with the selected architecture for the simulations in this study, mentioned in section 3.
- Abbreviations
  - MAE : Mean Absolute Error

Fourier coefficient selection

Number of Fourier coefficients

	1	3	5	7	9	11	13	15	17	23
**Loss**	0.0385	0.0056	0.0036	0.0032	0.0029	0.0028	0.0027	0.003	0.0031	0.0029
**Validation loss**	0.0403	0.006	0.0034	0.0029	0.0027	0.0026	0.0027	0.0025	0.0037	0.0032
**MAE**	0.1465	0.0474	0.04	0.0376	0.0353	0.0362	0.0354	0.0361	0.0382	0.0369
**Validation MAE**	0.1505	0.0481	0.0392	0.0362	0.0338	0.0347	0.0359	0.0327	0.0438	0.0421
